# Supplementary material for: Clinicopathologic and molecular predictors of survival in BRCA-deficient tubo-ovarian high-grade serous carcinoma
Source: Nat Commun. 2026 Apr 1;17:4789. doi: 10.1038/s41467-026-71134-3 (PMC13219410; doi:10.1038/s41467-026-71134-3)
Supplement: Supplementary file 1 — Supplementary Information [file 41467_2026_71134_MOESM1_ESM.pdf]

Clinicopathologic and molecular predictors of survival in *BRCA*-deficient tubo-ovarian high-grade serous carcinoma

Supplementary Information

Contents

1. Supplementary Figures

1.1. Supplementary Figure 1 | Patient cohorts and case selection: Overview for clinical, molecular, and validation analysis

1.2. Supplementary Figure 2 | *BRCA* status and residual disease as predictors of overall survival in HGSC (OTTA cohort) and progression-free survival (AOCS cohort)

1.3. Supplementary Figure 3 | Association of *BRCA* status and residual disease with distribution of molecular features in HGSC: Insights from the OTTA cohort

1.4. Supplementary Figure 4 | Association of *BRCA* status and neoadjuvant chemotherapy on survival in HGSC (AOCS cohort)

1.5. Supplementary Figure 5 | Distribution of pathogenic germline *BRCA1* and *BRCA2* variants by domain location, mutation type, and founder status in HGSC (AOCS cohort)

1.6. Supplementary Figure 6 | Distribution analysis of clinical and molecular features by *BRCA* and survival groups in HGSC (MOCOG cohort).

1.7. Supplementary Figure 7 | *NF1* gene alterations and expression (MOCOG cohort).

1.8. Supplementary Figure 8 | Survival analysis by *NF1* expression HGSC, with stratification by *BRCA* status: Findings from MOCOG and OTTA cohorts.

1.9. Supplementary Figure 9 | *PIK3CA* and *RAD21* gene alterations in HGSC: Findings from MOCOG and OTTA cohorts.

1.10. Supplementary Figure 10 | *c-KIT* gene expression in HGSC: Association with survival, molecular subtypes, and *BRCA* status (MOCOG cohort).

2. **Supplementary Tables**

2.1. Supplementary Table 1 | Univariable and multivariable AFT model analyses of overall survival in the AOCS cohort.

2.2. Supplementary Table 2 | Univariable and multivariable AFT model analyses of progression-free survival in the AOCS cohort.

2.3. Supplementary Table 3 | Multivariable AFT model of overall survival in the AOCS cohort excluding first-line PARP inhibitor maintenance therapy.

2.4. Supplementary Table 4 | Molecular and immune features stratified by residual disease and BRCA status in the AOCS cohort.

2.5. Supplementary Table 5 | Univariable and multivariable AFT model of overall survival by BRCA status and neoadjuvant chemotherapy in the AOCS cohort.

2.6. Supplementary Table 6 | Distribution of *CCNE1*, *NF1*, *PIK3CA*, *RAD21*, and *MYC* somatic alterations across BRCA-survival subgroups.

2.7. Supplementary Table 7 | Frequency of genes identified as significant in both differential methylation and differential expression analyses in the multi-omics cohort.

2.8. Supplementary Table 8 | Quartile odds ratios of immune cell subsets comparing short-term versus long-term survival in the multi-omics cohort.

3. **Supplementary Notes**

3.1. Definition of survival groups

3.2. Identification of *BRCA1* and *BRCA2* founder mutations

3.3. *BRCA*-deficiency

3.4. Sample quality control

3.5. Mutational signatures

3.6. NF1 immunohistochemistry analysis

3.7. Molecular subtype prediction

3.8. Clonality analysis

3.9. Mutual exclusivity

3.10. *MYC* amplification, RNA expression, and outcome in *BRCA2*-deficient HGSC

3.11. CIBERSORTx analysis

3.12. Methylation analysis

3.13. D11q analysis

3.14. Analysis of locus specific loss of heterozygosity in *NF1* alterations

3.15. Analysis of wildtype loss in *BRCA* mutated tumors

4. **Supplementary References**

1. Supplementary Figures

1.1. Supplementary Figure 1

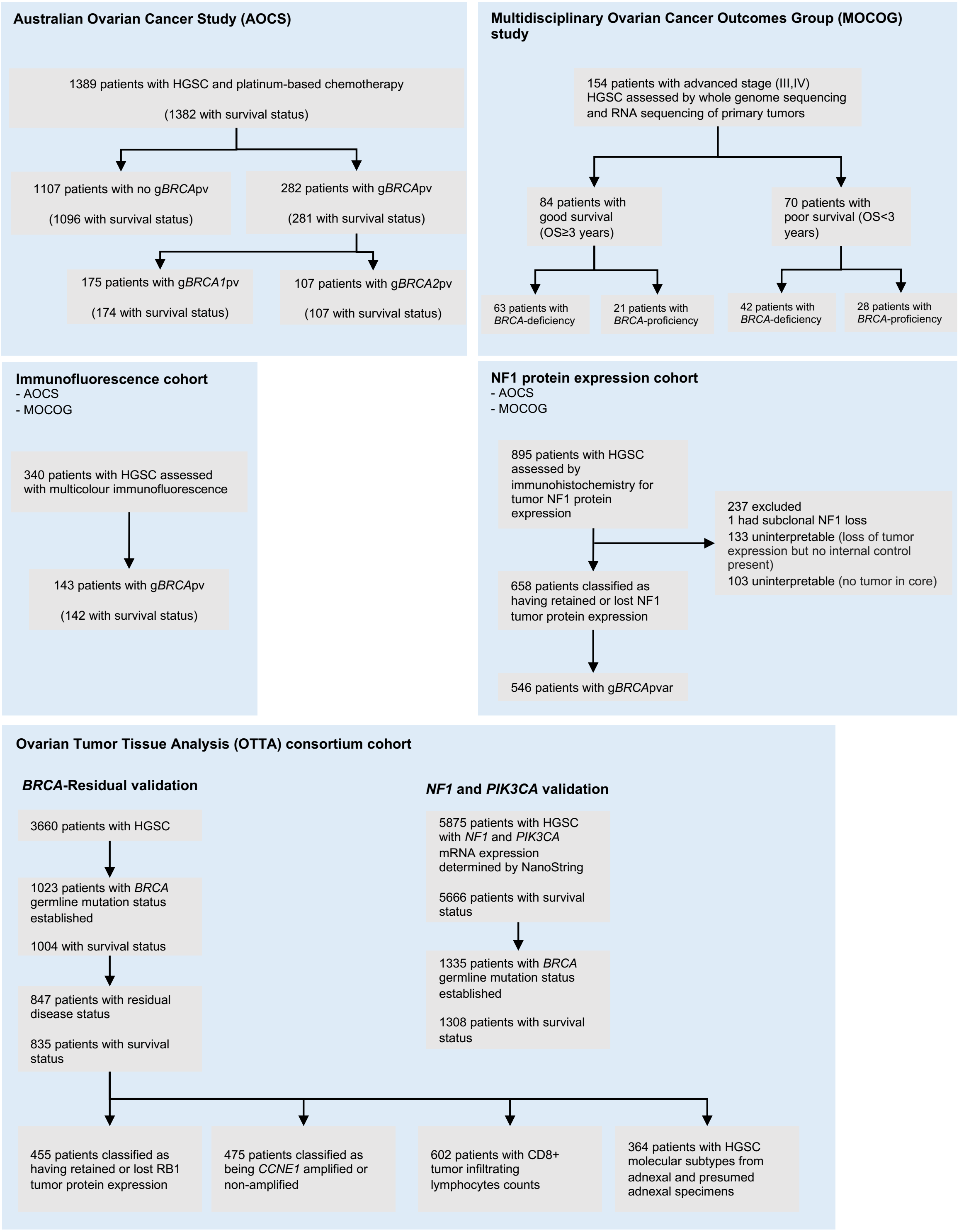

Supplementary Fig. 1 | Patient cohorts and case selection: Overview for clinical, molecular, and validation analysis. Overview of patient cohorts and case selection for the clinical, molecular and validation analysis. *HGSC=Tubo-ovarian high-grade serous carcinoma, AOCS =Australian Ovarian Cancer Study, MOCOG=Multidisciplinary Ovarian Cancer Outcome Group, gBRCApv=pathogenic germline BRCA variant, mcIF=multicolor immunofluorescence, OTTA = Ovarian Tumor Tissue Analysis, mRNA=messenger ribonucleic acid, OS=overall survival*

1.2. Supplementary Figure 2

A.

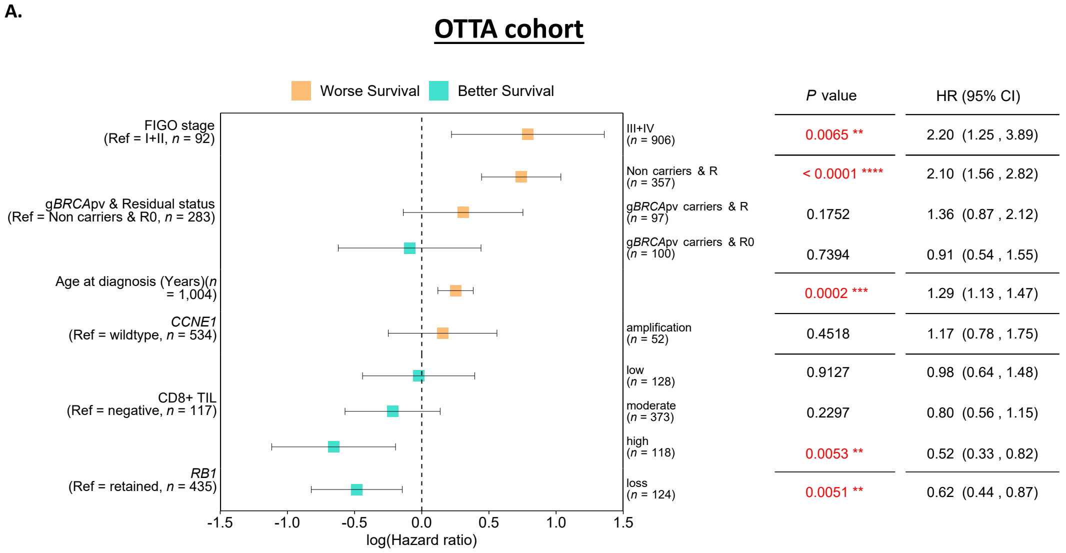

B.

AOCS cohort

	gBRCApv & R (n=174)	gBRCApv & R0 (n=105)	Non-carriers & R (n=649)	Non-carriers & R0 (n=356)
**Events**, n (%)	150 (86.2)	66 (66.9)	598 (92.1)	247 (69.4)
**Median PFS**, years	1.51	2.76	0.99	2.23
**5-Year PFS rate**, %	16.7	37.5	7.3	30.1

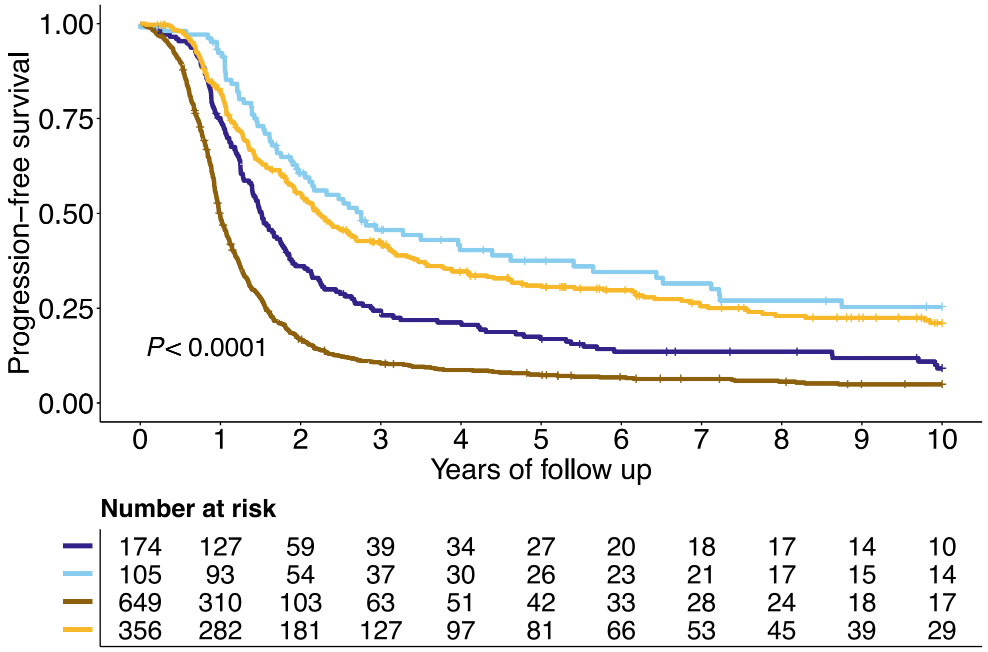

Supplementary Fig. 2 | *BRCA* status and residual disease as predictors of overall survival in HGSC (OTTA cohort) and progression-free survival (AOCS cohort). a, Multivariable Cox proportional hazards model of the interaction term *BRCA* and residual disease status and clinicopathological and molecular predictive features on overall survival with patients (n = 1004) from the OTTA cohort. *P* values were derived using the Wald test; values < 0.05 are colored red (*, $P < 0.05$; **, $P < 0.01$; ***, $P < 0.001$; ****$P < 0.0001$). **b**, Kaplan-Meier curve of progression-free survival for the interaction term *BRCA* and Residual status from patients (n = 1284) of the Australian Ovarian Cancer Study (AOCS) cohort. *P* values calculated by log-rank test. Distribution of molecular features (CD8+ TIL density, molecular subtypes, and *RB1* loss) within the *BRCA* and residual groups by odds ratios. *P*-values were calculated based on odds ratios (OR). *R=Residual disease, R0=No residual disease, gBRCApv=pathogenic germline BRCA variant, HR=Hazard ratio, CI=confidence interval, n=Number of patients.*

1.3. Supplementary Figure 3

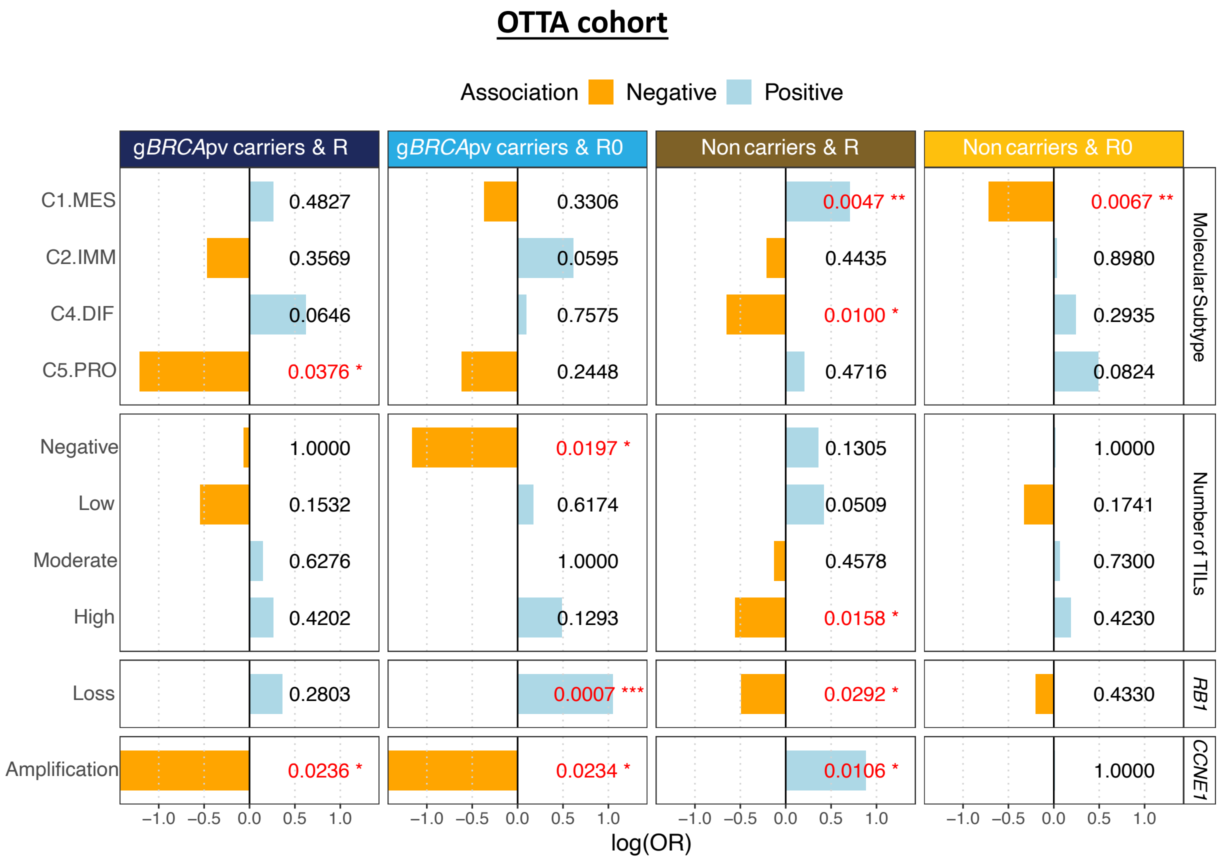

Supplementary Fig. 3 | Association of *BRCA* status and residual disease with distribution of molecular features in HGSC: Insights from the OTTA cohort. Distribution of molecular features (CD8+ TIL density, molecular subtypes, and *RB1* loss) within the *BRCA* and residual groups (n = 835 patients) by odds ratios. *P*-values were calculated based on odds ratios (OR). Source data are provided as a Source Data file. *R=Residual disease, R0=No residual disease, gBRCApv=pathogenic germline BRCA variant, , TIL= tumor-infiltrating lymphocyte*

1.4. Supplementary Figure 4

AOCS cohort

	g*BRCA*pv & no NACT (n=220)	g*BRCA*pv & NACT (n=54)	Non-carriers & no NACT (n=828)	Non-carriers & NACT (n=268)
**Events**, n (%)	123 (55.9)	31 (57.1)	598 (72.2)	170 (63.4)
**Median OS**, years	6.21	4.46	3.80	3.10
**5-Year OS rate**, %	57.8	43.1	40.5	30.9

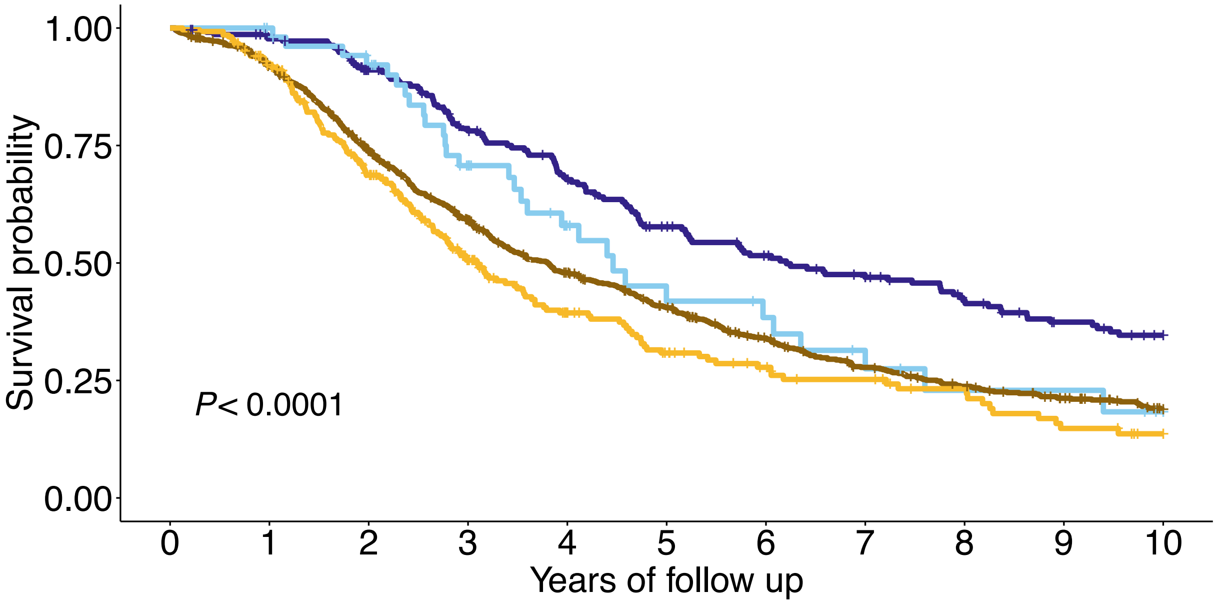

Number at risk

	0	1	2	3	4	5	6	7	8	9	10
g*BRCA*pv & no NACT	220	210	185	154	130	106	91	79	66	54	46
g*BRCA*pv & NACT	54	52	46	31	19	13	11	7	5	5	2
Non-carriers & no NACT	828	741	579	449	354	288	222	174	145	120	93
Non-carriers & NACT	268	237	165	100	63	45	34	28	22	14	9

Supplementary Fig. 4 | Association of *BRCA* status and neoadjuvant chemotherapy on survival in HGSC (AOCS cohort). Kaplan-Meier analysis of overall survival stratified by the interaction term *BRCA* and neoadjuvant chemotherapy status from patients (n=1370) of the Australian Ovarian Cancer Study (AOCS) cohort. Source data are provided as a Source Data file. *P* value calculated by log-rank test. *gBRCApv=pathogenic germline BRCA variant, NACT=neoadjuvant chemotherapy, n=Number of patients, OS=Overall survival*

1.5. Supplementary Figure 5

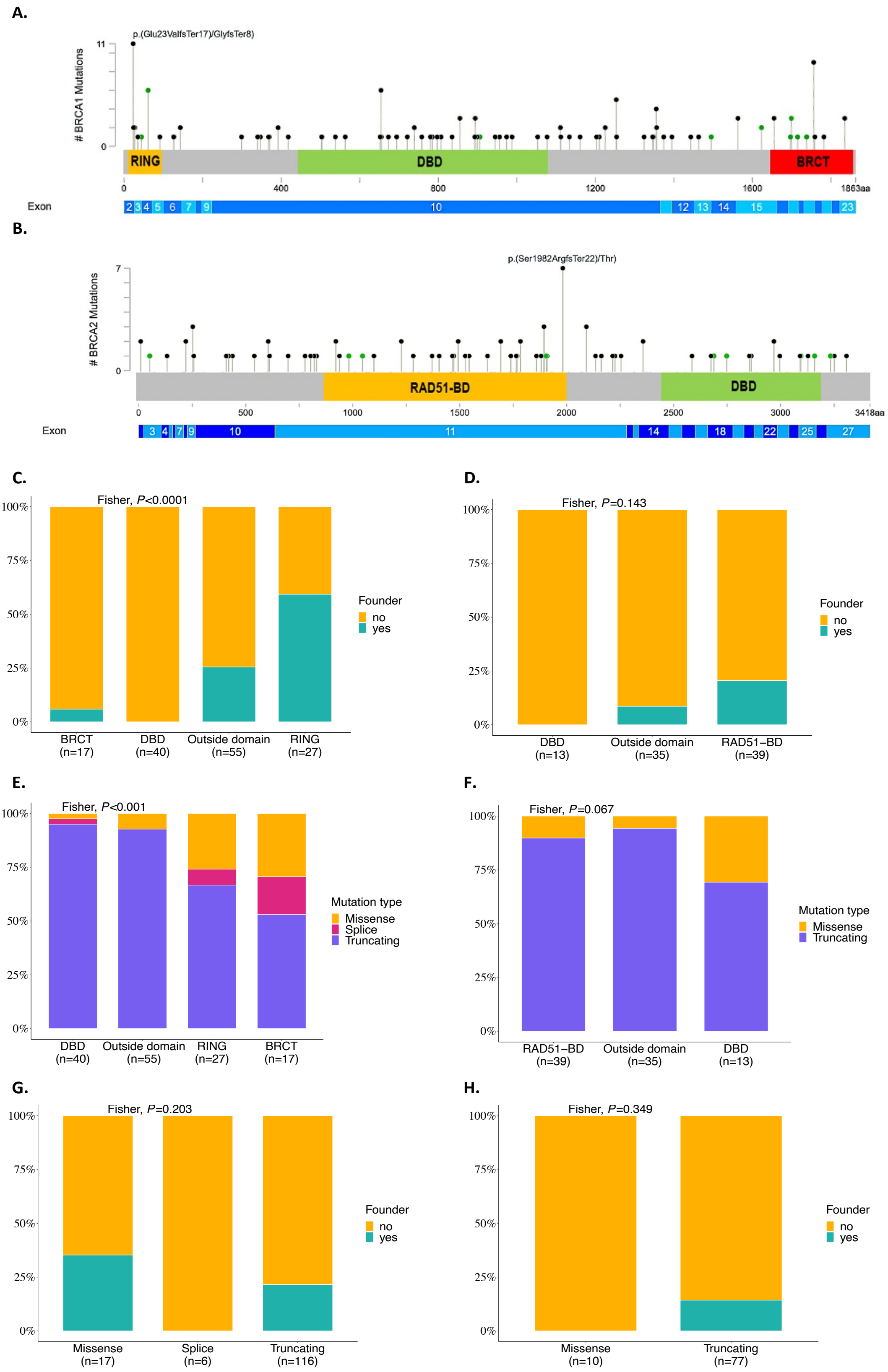

Supplementary Fig. 5 | Distribution of pathogenic germline *BRCA1* and *BRCA2* variants by domain location, mutation type, and founder status in HGSC (AOCS cohort). a, and **b**, shows the distribution of pathogenic germline mutations on the *BRCA1* (n = 139) and *BRCA2* (n = 87*)* gene, respectively. **c**, and **d**, show the distribution of founder mutations across functional domains of *BRCA1* (n = 139) (**c**) and *BRCA2* (n = 87) (**d**). **e**, and **f**, show the distribution of mutation types within the different *BRCA1* (n = 139) (**e**) and *BRCA2* (n = 87) (**f**) functional domains. **g**, and **h**, show the distribution of founder mutations across mutation types of *BRCA1* (n = 139) (**g**) and *BRCA2* (n = 87) (**h**). Fisher's exact test *P* value is reported. Source data are provided as a Source Data file. *gBRCApv=pathogenic germline BRCA variant, DBD=DNA binding domain, RING=Really Interesting New Gene domain, RAD51-BD=RAD51-binding domain, BRCT=BRCA c-terminal domain, n=Number of patients*

1.6. Supplementary Figure 6

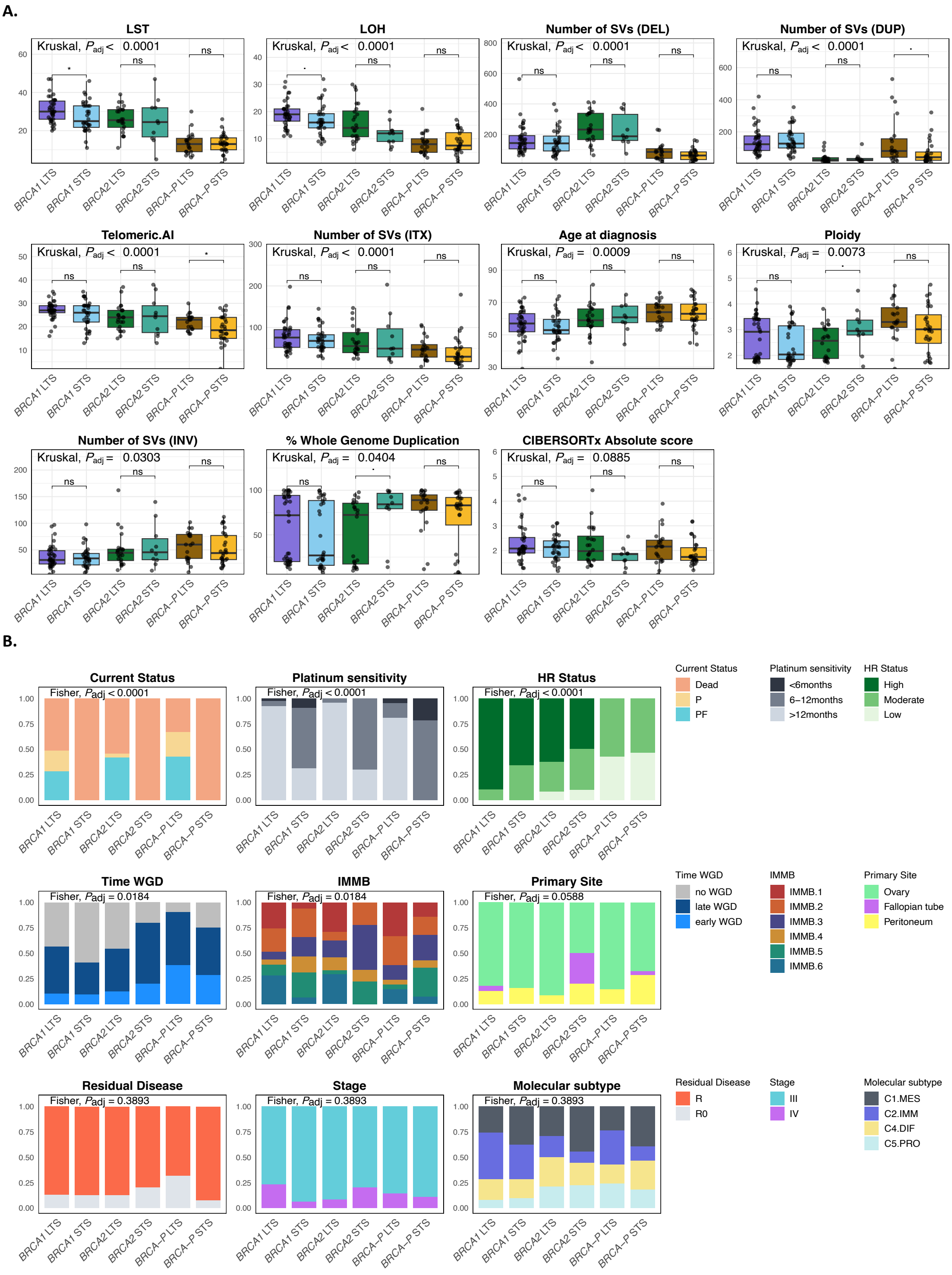

Supplementary Fig. 6 | Distribution analysis of clinical and molecular features by *BRCA* and survival groups in HGSC (MOCOG cohort). a, Box plots summarizing numerical, clinical and genomic characteristics by *BRCA* and survival groups (n = 154 patients); dots represent each sample, boxes show the interquartile range (25-75th percentiles), central lines show the median, whiskers show the smallest/largest values within 1.5 times the interquartile range. Kruskal-Wallis test Benjamini-Hochberg adjusted *P*-values and pairwise Mann-Whitney-Wilcoxon test *P* values (two sided) are reported (ns, P>0.1; ·, P<0.1; *, P < 0.05; **, P < 0.01; ***, P < 0.001). Features are ranked according to their significance. **b**, Proportion of patients with categorical characteristics per *BRCA* and survival group (n = 154 patients). Features are ordered by significance using Fisher's exact test (two-tailed) and clusters are ordered by proportion of long-term survivors. Fisher's test *P*-values shown are Benjamini-Hochberg adjusted *P*-values. Features are ordered by significance. Source data are provided as a Source Data file. *LST=Large scale transitions, LOH= Loss of heterozygosity, SV= Structural variants, DEL=Deletion, DUP=Duplication, AI=Allelic imbalance, INV=Inversion; Long term survivor (LTS)= OS >3 years, Short term survivor (STS)= OS ≤3 years, BRCA-P=BRCA-proficient; HR status= Homologous recombination status, P=Progression, PF= Progression-free; R=residual disease, R0=no residual disease; High ≥63, Moderate= 42-62, Low= ≤41, IMMB=Immune clusters (by CIBERSORTx), Molecular subtypes: C1.MES=C1 mesenchymal subtype, C2.IMM=C2 immunoreactive subtype, C4.DIF=C4 differentiated subtype, C5.PRO=C5 proliferative subtype*

1.7. Supplementary Figure 7

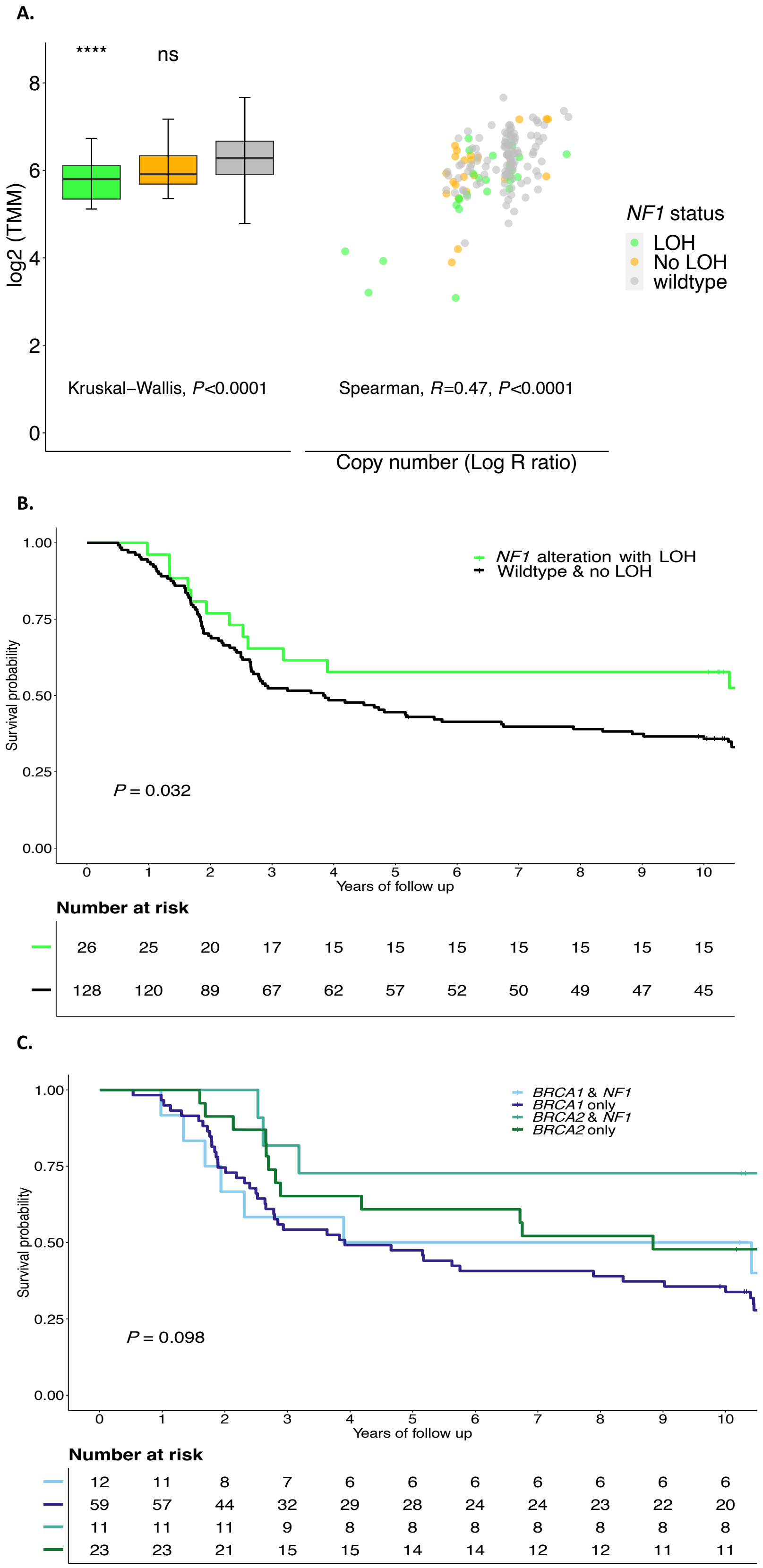

Extended Data Fig. 7 | *NF1* gene alterations and expression (MOCOG cohort). a, Scatter graphs (right) show *NF1* expression (y-axis) plotted against copy number (x-axis) in primary tumors (n=153, Spearman correlation analysis). Boxplots (left) summarize *NF1* expression by *NF1* alterations with and without locus specific loss of heterozygosity (LOH); lines indicate median, and whiskers show range. Kruskal–Wallis test *P* value is reported as well as pairwise Wilcoxon rank-sum test *P* values comparing altered groups to wildtype (non-significant (ns), P>0.05; ****, P <0.0001). **b**, Kaplan-Meier analysis of overall survival in patients (n = 154) stratified by *NF1* alterations exhibiting locus specific LOH and **c**, in patients (n = 105) with *BRCA1*-and *BRCA2*-deficient tumors stratified by *NF1* alteration status. *P* values calculated by log-rank test. Source data are provided as a Source Data file. *TMM=Trimmed Mean of M-values*

1.8. Supplementary Figure 8

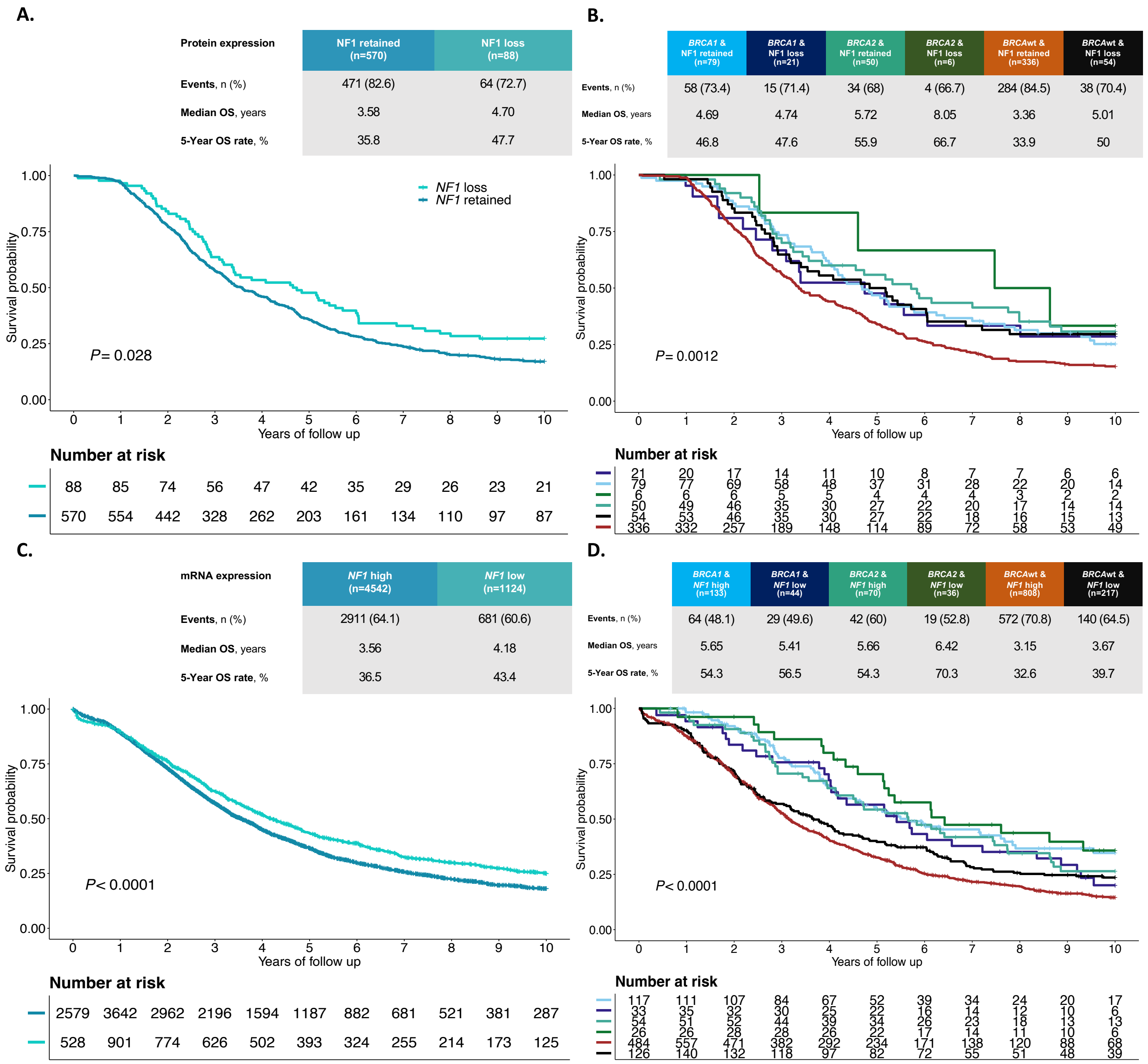

Supplementary Fig. 8 | Survival analysis by *NF1* expression HGSC, with stratification by *BRCA* status: Findings from MOCOG and OTTA cohorts. Kaplan-Meier curves for overall survival (OS) comparing **a,** patients (n = 658) with HGSC from the MOCOG cohort by NF1 protein expression status (NF1 retained vs loss) and in **b,** additionally stratified by germline *BRCA* mutation status (n = 546 patients). **c**, Kaplan-Meier curve comparing the overall survival of patients (n = 5666) with HGSC from the OTTA cohort by *NF1* RNA expression status (low=lowest quantile, high=2nd to 5th quantiles) and in **d,** additionally stratified by germline *BRCA* mutation status (n = 1308 patients). *P* values calculated by log-rank test. Source data are provided as a Source Data file.

1.9. Supplementary Figure 9

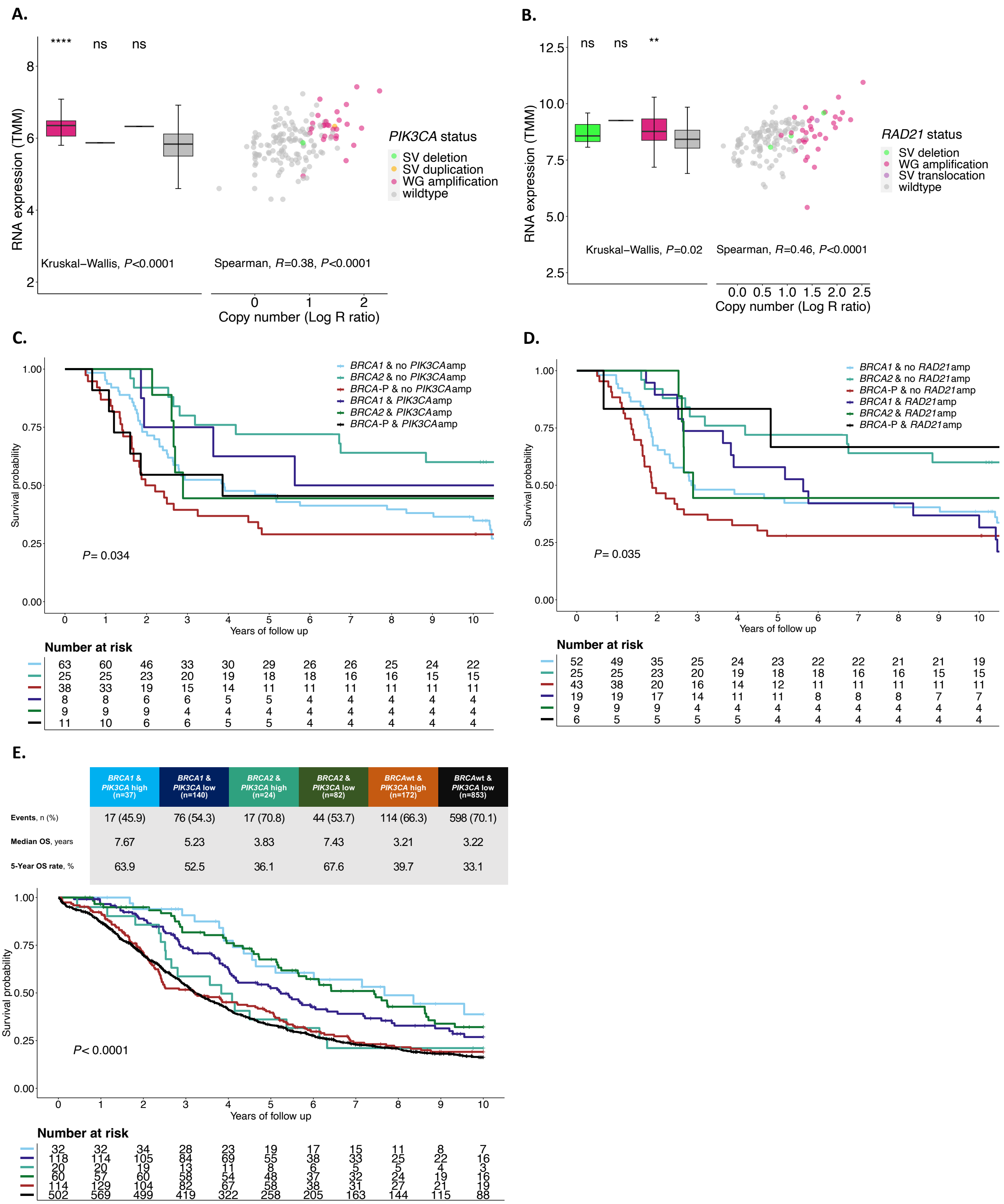

	BRCA1 & PIK3CA high (n=37)	BRCA1 & PIK3CA low (n=140)	BRCA2 & PIK3CA high (n=24)	BRCA2 & PIK3CA low (n=82)	BRCAwt & PIK3CA high (n=172)	BRCAwt & PIK3CA low (n=853)
Events, n (%)	17 (45.9)	76 (54.3)	17 (70.8)	44 (53.7)	114 (66.3)	598 (70.1)
Median OS, years	7.67	5.23	3.83	7.43	3.21	3.22
5-Year OS rate, %	63.9	52.5	36.1	67.6	39.7	33.1

Supplementary Fig. 9 | *PIK3CA* and *RAD21* gene alterations in HGSC: Findings from MOCOG and OTTA cohorts. a, **b**, Scatter graphs (right) of the expression (y-axis) of *PIK3CA* (a) and *RAD21* (b) plotted against copy number (x-axis) in primary tumors (n=153, Spearman correlation analysis). Boxplots (left) summarize expression by mutation type; lines indicate median, and whiskers show range. Kruskal–Wallis test *P* value is reported as well as pairwise Wilcoxon rank-sum test *P* values comparing altered groups to wildtype (non-significant (ns), *P*>0.05; ****, *P* <0.0001; ***, *P* <0.001; **, *P* <0.01). **c**, Kaplan-Meier analysis of overall survival in patients (n = 154) with HGSC stratified by *BRCA*-status and *PIK3CA* amplification vs no amplification. *P* value calculated by log-rank test. **d**, Kaplan-Meier analysis of overall survival in patients (n = 154) with HGSC stratified by *BRCA*-status and *RAD21* amplification vs no amplification. *P* value calculated by log-rank test. **e**, Kaplan-Meier analysis of overall survival in patients (n = 846) with HGSC from the OTTA cohort stratified by *PIK3CA* RNA expression status (high=highest quantile, low=1st to 4th quantiles) and stratified by germline *BRCA* mutation status. *P* value calculated by log-rank test. Source data are provided as a Source Data file. *SV=Structural variants, amp=amplification, WG=Whole gene, BRCA-P=BRCA-proficient, TMM=Trimmed Mean of M-values*

1.10. Supplementary Figure 10

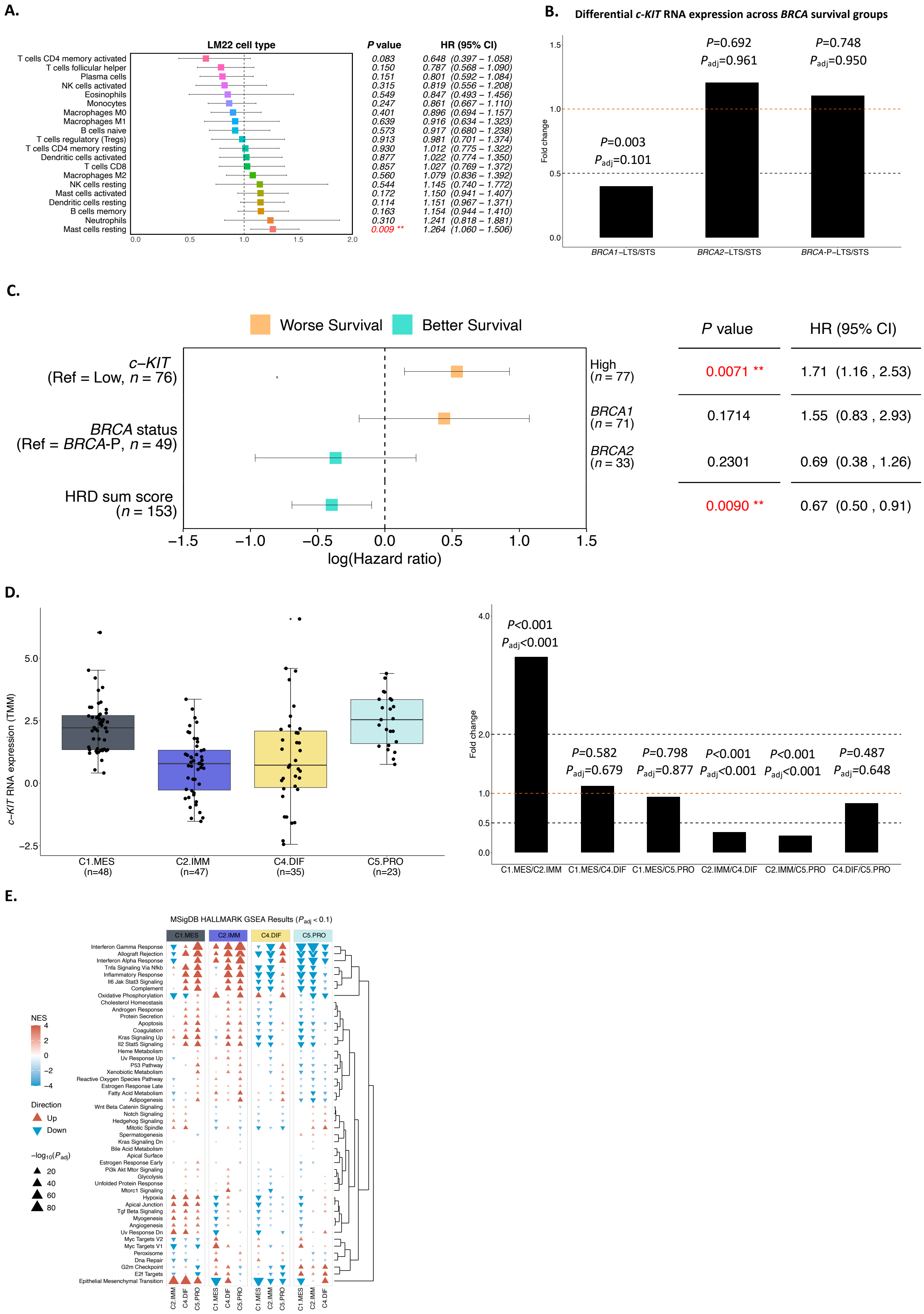

Supplementary Fig. 10 | *c-KIT* gene expression in HGSC: Association with survival, molecular subtypes, and *BRCA* status (MOCOG cohort). a, Forest plot (left) indicates the hazard ratio (HR, squares) and 95% confidence

interval (CI; whiskers) for overall survival (OS) calculated using a multivariable Cox proportional hazard regression model based on the LM22 immune cell types detected by CIBERSORTx analysis (n = 153 patients). Cell types are arranged by HR. *P* values were derived by Wald test; values < 0.05 are colored red (**P* < 0.05, ***P* < 0.01). **b**, Differential expression analysis was performed using DESeq2 to determine fold change (right) of gene expression between the *BRCA* survival groups (*BRCA1=BRCA1*-deficient; *BRCA2=BRCA2*-deficient; *BRCA-P*=*BRCA*-proficient; Long term survivor (LTS) = OS >3 years; Short term survivor (STS) = OS ≤3 years) (n = 153 patients) (two-tailed Wald test, both unadjusted *P* values and Benjamini-Hochberg adjusted *P* values (P_{adj}) are shown). **c**, Multivariable Cox proportional hazards model for OS comparing *c-KIT* with high vs low RNA expression levels by median and adjusted for HRD sum score and BRCA HRD status (n = 153 patients). *P* values were derived by Wald test; values < 0.05 are colored red (**P* < 0.05, ***P* < 0.01, ****P* < 0.001, *****P* < 0.0001). **d**, Boxplots summarize RNA expression of the *c-KIT* gene marker across the molecular subtypes (C1.MES=C1 mesenchymal subtype, C2.IMM=C2 immunoreactive subtype, C4.DIF=C4 differentiated subtype, C5.PRO=C5 proliferative subtype) (n = 153 patients); points represent each sample, boxes show the interquartile range (25–75th percentiles), central lines indicate the median, and whiskers show the smallest/largest values within 1.5 times the interquartile range. Differential expression analysis was performed using DESeq2 to determine fold change (right) of gene expression between the molecular subtypes (two-tailed Wald test, both unadjusted *P* values and Benjamini-Hochberg adjusted *P* values (P_{adj}) are shown). **e**, Clustered heatmap summarizing gene set enrichment analysis (GSEA) using the hallmark Molecular Signatures Database (MSigDB) gene sets (n = 153 patients). Direction and color of triangles relate to the normalized enrichment score (NES) as generated by FGSEA. *P* values (two-sided) were calculated using the FGSEA default Monte Carlo method; the size of the triangles corresponds to the negative $\log_{10}$ Benjamini-Hochberg adjusted *P* value (P_{adj}). Columns are separated by molecular subtypes with the direction of enrichment indicated by the first group mentioned in the x-axis label. Source data are provided as a Source Data file. *BRCA-P= BRCA-proficient, Survival group: Long-term survivor (LTS)= OS >3 years, Short-term survivor (STS)= OS ≤3 years, TMM=Trimmed Mean of M-values*

2. Supplementary Tables

2.1. Supplementary Table 1

Univariable and multivariable AFT model analyses of overall survival in the AOCS cohort. Univariable and multivariable Accelerated Failure Time (AFT) model results of clinical features, including interaction analyses with germline *BRCA* pathogenic variant (g*BRCA*pv) status, on overall survival in patients (n = 1377) with HGSC from the Australian Ovarian Cancer Study (AOCS) cohort. Time ratios (TR) and 95% confidence intervals (CI) were estimated using a log-logistic AFT model. A TR > 1 indicates a longer survival time; a TR < 1 indicates shorter survival. Two-sided *P*-values were derived from Wald tests. Interaction *P*-values indicate whether the association between each clinical feature and survival differs by *BRCA* status. Source data are provided as a Source Data file.

			Univariable					Multivariable				
				95%CI					95%CI			
**Feature**	**Factor**	**Number**	**TR**	**lower**	**upper**	***P*-value ***	***P*-value****	**TR**	**lower**	**upper**	***P*-value***	***P*-value****
BRCA-status	Non-carriers	1096	-	-	-	-	-	-	-	-	-	-
	g*BRCA*pv-carriers	281	1.72	1.5	1.98	<0.001	-	1.53	1.33	1.76	<0.001	-
Residual disease	R0	461	-	-	-	-	-	-	-	-	-	-
	R	823	0.46	0.41	0.52	<0.001	0.008	0.55	0.48	0.62	<0.001	0.011
FIGO stage	I+II	134	-	-	-	-	-	-	-	-	-	-
	III+IV	1183	0.34	0.28	0.42	<0.001	0.04	0.59	0.47	0.74	<0.001	0.121
Primary site	Ovary	1002	-	-	-	-	-	-	-	-	-	-
	FT	135	1.28	1.04	1.58	0.023	0.544	1.1	0.89	1.36	0.372	0.138
	Peritoneum	215	0.66	0.57	0.77	<0.001	0.872	0.82	0.71	0.94	0.006	0.836
Age at diagnosis	Years Spline 1	1370	0.99	0.98	1	0.069	0.082	1	0.99	1.01	0.625	0.188
	Years Spline 2		0.99	0.97	1	0.142	0.213	0.98	0.97	1	0.027	0.201
Surgery	Primary CS	980	-	-	-	-	-	-	-	-	-	-
	Interval CS	299	0.83	0.72	0.95	0.007	0.675	0.84	0.45	1.56	0.586	0.507
	Other	69	1.27	0.99	1.63	0.065	0.697	1.13	0.83	1.53	0.44	0.743
Neoadjuvant CHT	No	1048	-	-	-	-	-	-	-	-	-	-
	Yes	322	0.83	0.72	0.95	0.006	0.563	1	0.54	1.84	0.999	0.12
Grade	G2	237	-	-	-	-	-	-	-	-	-	-
	G3	1088	1.22	1.05	1.41	0.009	0.907	1.06	0.92	1.21	0.411	0.356
PARP inhibitor	No	1338	-	-	-	-	-	-	-	-	-	-
	Yes	39	1.4	0.92	2.12	0.119	0.347	1.24	0.8	1.92	0.332	0.524

**Wald-test*
*** interaction with BRCA status*
R=Residual disease, R0=No residual disease, G2=Grade 2, G3=Grade 3, OS=Overall survival, gBRCApv=pathogenic germline BRCA variant, TR=Time ratio, CI=confidence interval, CHT=chemotherapy, CS=cytoreductive surgery, FT=fallopian tube

2.2. Supplementary Table 2

Univariable and multivariable AFT model analyses of progression-free survival in the AOCS cohort. Univariable and multivariable Accelerated Failure Time (AFT) model results of clinical features, including interaction analyses with germline *BRCA* pathogenic variant (g*BRCA*pv) status, on progression-free survival in patients (n = 1377) with HGSC from the Australian Ovarian Cancer Study (AOCS) cohort. Time ratios (TR) and 95% confidence intervals (CI) were estimated using a log-logistic AFT model. A TR > 1 indicates a longer survival time; a TR < 1 indicates shorter survival. Two-sided *P*-values were derived from Wald tests. Interaction *P*-values indicate whether the association between each clinical feature and survival differs by BRCA status. Source data are provided as a Source Data file.

			Univariable					Multivariable				
				95%CI					95%CI			
**Feature**	**Factor**	**Number**	**TR**	**lower**	**upper**	***P*-value ***	***P*-value****	**TR**	**lower**	**upper**	***P*-value***	***P*-value****
BRCA-status	Non-carriers	1096	-	-	-	-	-	-	-	-	-	-
	g*BRCA*pv-carriers	281	1.53	1.34	1.76	<0.001	-	1.34	1.28	1.53	<0.001	-
Residual disease	R0	461	-	-	-	-	-	-	-	-	-	-
	R	823	0.45	0.4	0.5	<0.001	0.109	0.57	0.5	0.64	<0.001	0.042
FIGO stage	I+II	134	-	-	-	-	-	-	-	-	-	-
	III+IV	1183	0.25	0.2	0.31	<0.001	0.195	0.41	0.33	0.52	<0.001	0.353
Primary site	Ovary	1002	-	-	-	-	-	-	-	-	-	-
	FT	135	1.27	1.04	1.54	0.019	0.359	1.01	0.84	1.22	0.902	0.021
	Peritoneum	215	0.65	0.57	0.75	<0.001	0.99	0.81	0.71	0.93	0.002	0.952
Age at diagnosis	Years Spline 1	1370	0.99	0.97	1	0.069	0.06	1	0.98	1.01	0.479	0.188
	Years Spline 2		1	0.99	1.02	0.797	0.149	1	0.99	1.01	0.949	0.201
Surgery	Primary CS	980	-	-	-	-	-	-	-	-	-	-
	Interval CS	299	0.74	0.65	0.83	<0.001	0.878	0.9	0.52	1.56	0.719	0.911
	Other	69	1.14	0.88	1.47	0.315	0.853	1.13	0.85	1.5	0.413	0.424
Neoadjuvant CHT	No	1048	-	-	-	-	-	-	-	-	-	-
	Yes	322	0.73	0.64	0.82	<0.001	0.678	0.83	0.49	1.42	0.501	0.512
Grade	G2	237	-	-	-	-	-	-	-	-	-	-
	G3	1088	1.24	1.07	1.43	0.004	0.431	1.04	0.91	1.28	0.565	0.361
PARP inhibitor	No	1338	-	-	-	-	-	-	-	-	-	-
	Yes	39	1.87	1.3	2.7	0.001	0.627	1.84	1.26	2.68	0.002	0.791

**Wald-test*
*** interaction with BRCA status*
R=Residual disease, R0=No residual disease, G2=Grade 2, G3=Grade 3, OS=Overall survival, gBRCApv=pathogenic germline BRCA variant, TR=Time ratio, CI=confidence interval, CHT=chemotherapy, CS=cytoreductive surgery, FT=fallopian tube

2.3. Supplementary Table 3

Multivariable AFT model of overall survival in the AOCS cohort excluding first-line PARP inhibitor maintenance therapy. Multivariable Accelerated Failure Time (AFT) model of *BRCA* and residual disease status and clinicopathological predictive features on overall survival in patients (n = 1338) from the Australian Ovarian Cancer Study (AOCS) cohort excluding patients receiving first-line PARP inhibitor maintenance therapy. Time ratios (TR) and 95% confidence intervals (CI) were estimated using a log-logistic AFT model. A TR > 1 indicates a longer survival time; a TR < 1 indicates shorter survival. Two-sided *P*-values were derived from Wald tests. Source data are provided as a Source Data file.

			Univariable				Multivariable			
				95%CI				95%CI		
**Feature**	**Factor**	**Number**	**TR**	**lower**	**upper**	***P*-value ***	**TR**	**lower**	**upper**	***P*-value***
g*BRCA*pv & Residual status	Non carriers & R0	350	-	-	-	-	-	-	-	-
	Non carriers & R	642	0.42	0.37	0.48	<0.001	0.51	0.44	0.58	<0.001
	g*BRCA*pv carriers & R0	91	1.27	1	1.62	0.053	1.16	0.91	1.49	0.232
	g*BRCA*pv carriers & R	163	0.8	0.67	0.97	0.019	0.87	0.71	1.05	0.142
FIGO stage	I+II	131	-	-	-	-	-	-	-	-
	III+IV	1149	0.34	0.28	0.42	<0.001	0.59	0.47	0.74	<0.001
Primary site	Ovary	978	-	-	-	-	-	-	-	-
	FT	129	1.28	1.03	1.59	0.024	1.11	0.89	1.37	0.347
	Peritoneum	212	0.67	0.57	0.77	<0.001	0.81	0.7	0.94	0.006
Age at diagnosis	Years Spline 1	1331	0.99	0.98	1	0.086	1	0.99	1.01	0.61
	Years Spline 2		0.99	0.97	1	0.136	0.98	0.97	1	0.024
Surgery	Primary CS	960	-	-	-	-	-	-	-	-
	Interval CS	283	0.82	0.72	0.95	0.006	0.89	0.47	1.71	0.734
	Other	66	1.32	1.01	1.71	0.038	1.13	0.84	1.54	0.416
Neoadjuvant CHT	No	1026	-	-	-	-	-	-	-	-
	Yes	305	0.82	0.72	0.94	0.004	0.96	0.51	1.81	0.895
Grade	G2	237	-	-	-	-	-	-	-	-
	G3	1049	1.21	1.04	1.4	0.012	1.07	0.93	1.22	0.341

**Wald-test*
R=Residual disease, R0=No residual disease, G2=Grade 2, G3=Grade 3, OS=Overall survival, gBRCApv=pathogenic germline BRCA variant, TR=Time ratio, CI=confidence interval, CHT=chemotherapy, CS=cytoreductive surgery, FT=fallopian tube

2.4. Supplementary Table 4

Molecular and immune features stratified by residual disease and BRCA status in the OTTA cohort. Distribution of molecular subtypes, tumor-infiltrating lymphocytes, RB1 protein expression status, and *CCNE1* amplification status stratified by residual disease or no residual disease, and by germline BRCA pathogenic variant (g*BRCA*pv) carrier or non-carrier status, in patients (n = 847) from the Ovarian Tumor Tissue Association (OTTA) cohort. Percentages are given in parentheses and calculated within each subgroup. Source data are provided as a Source Data file.

	**g*BRCA*pv carriers & R** (n, %)	**g*BRCA*pv carriers & R0** (n, %)	**Non carriers & R** (n, %)	**Non carriers & R0** (n, %)
**Molecular Subtype**	(n=48)	(n=56)	(n=132)	(n=129)
C1.MES	15 (31.25%)	12 (21.43%)	48 (36.36%)	24 (18.60%)
C2.IMM	9 (18.75%)	19 (33.93%)	28 (21.21%)	31 (24.03%)
C4.DIF	21 (43.75%)	19 (33.93%)	31 (23.48%)	46 (35.66%)
C5.PRO	3 (6.25%)	6 (10.71%)	25 (18.94%)	28 (21.71%)
**Number of TILs**	(n=79)	(n=71)	(n=253)	(n=201)
Negative	12 (15.19%)	4 (5.63%0	44 (17.39%)	30 (14.93%)
Low	9 (11.39%)	14 (19.72%)	53 (20.95%)	29 (14.43%)
Moderate	42 (53.16%)	36 (50.70%)	124 (49.01%)	104 (51.74%)
High	16 (20.25%)	17 (33.15%)	32 (12.65%)	38 (18.91%)
**RB1**	(n=44)	(n=55)	(n=190)	(n=166)
Retained	30 (68.18%)	25 (45.45%)	38 (20%)	38 (22.89%)
Loss	14 (31.82%)	30 (54.55%)	152 (80%)	128 (77.11%)
CCNE1	(n=47)	(n=49)	(n=205)	(n=174)
No Amplification	0 (0%)	0 (0%)	181 (88.29%)	160 (91.95%)
Amplification	47 (100%)	49 (100%)	24 (11.71%)	14 (8.05%)

TILs= Tumor-infiltrating lymphcytes, R=Residual disease, R0=No residual disease, gBRCApv=pathogenic germline BRCA variant, C1.MES=C1 mesenchymal subtype, C2.IMM=C2 immunoreactive subtype, C4.DIF=C4 differentiated subtype, C5.PRO=C5 proliferative subtype

2.5. Supplementary Table 5

Univariable and multivariable AFT model of overall survival by BRCA status and neoadjuvant chemotherapy in the AOCS cohort. Univariable and multivariable Accelerated Failure Time (AFT) model of *BRCA* and neoadjuvant chemotherapy status and clinicopathological predictive features on overall survival in patients (n = 1370) from the Australian Ovarian Cancer Study (AOCS) cohort. The model was fitted using a log-logistic distribution. Results are expressed as Time Ratios (TR) with corresponding 95% confidence intervals (CI). Two-sided *P*-values were derived from Wald tests. A TR > 1 indicates a longer survival time, whereas a TR < 1 indicates a shorter survival time. Age at diagnosis was modeled using restricted cubic splines with 3 knots and is presented as two spline terms. Source data are provided as a Source Data file.

			**Univariable**				**Multivariable**			
				**95%CI**				**95%CI**		
**Feature**	**Factor**	**Number**	**TR**	**lower**	**upper**	***P*-value ***	**TR**	**lower**	**upper**	***P*-value***
g*BRCA*pv & NACT status	Non carriers & no NACT	828	-	-	-	-	-	-	-	-
	Non carriers & NACT	268	0.85	0.74	0.99	0.034	1.17	0.62	2.21	0.634
	g*BRCA*pv carriers & no NACT	220	1.7	1.46	2	<0.001	1.6	1.37	1.87	<0.001
	g*BRCA*pv carriers & NACT	54	1.32	0.99	1.75	0.06	1.39	0.75	2.6	0.298
Residual disease	R0	461	-	-	-	-	-	-	-	-
	R	823	0.46	0.41	0.52	<0.001	0.54	0.48	0.62	<0.001
FIGO stage	I+II	134	-	-	-	-	-	-	-	-
	III+IV	1183	0.34	0.28	0.42	<0.001	0.59	0.47	0.74	<0.001
Primary site	Ovary	1002	-	-	-	-	-	-	-	-
	FT	135	1.28	1.04	1.58	0.023	1.1	0.89	1.35	0.392
	Peritoneum	215	0.66	0.57	0.77	<0.001	0.81	0.7	0.94	0.005
Age at diagnosis	Years Spline 1	1370	0.99	0.98	1	0.069	1	0.99	1.01	0.694
	Years Spline 2		0.99	0.97	1	0.142	0.98	0.97	1	0.034
Surgery	Primary CS	980	-	-	-	-	-	-	-	-
	Interval CS	299	0.83	0.72	0.95	0.007	0.75	0.4	1.42	0.376
	Other	69	1.27	0.99	1.63	0.065	1.12	0.83	1.52	0.46
Grade	G2	237	-	-	-	-	-	-	-	-
	G3	1088	1.22	1.05	1.41	0.009	1.06	0.92	1.21	0.415
PARP inhibitor	No	1338	-	-	-	-	-	-	-	-
	Yes	39	1.4	0.92	2.12	0.119	1.28	0.82	1.98	0.273

**Wald-test*
R=Residual disease, R0=No residual disease, G2=Grade 2, G3=Grade 3, OS=Overall survival, gBRCApv=pathogenic germline BRCA variant, TR=Time ratio, CI=confidence interval, NACT=Neoadjuvant chemotherapy, CS=cytoreductive surgery, FT=fallopian tube

2.6. Supplementary Table 6

Distribution of *CCNE1*, *NF1*, *PIK3CA*, *RAD21*, and *MYC* somatic alterations across BRCA-survival subgroups. Pairwise Fisher's exact tests assessing the distribution of *CCNE1*, *NF1*, *PIK3CA*, *RAD21*, and *MYC* alterations across *BRCA*-survival subgroups in the multi-omics cohort (n = 154 patients). This table summarises unadjusted and Benjamini-Hochberg-adjusted *P*-values from two-sided pairwise Fisher's exact tests evaluating whether the frequency of *CCNE1*, *NF1*, *PIK3CA*, *RAD21*, or *MYC* somatic alterations differs between *BRCA1*, *BRCA2*, and *BRCA*-proficient long-term survivors (LTS) and short-term survivors (STS). Each comparison tests whether the proportion of altered versus non-altered cases differs between two contrasting BRCA-survival groups. No additional filtering criteria were applied beyond inclusion of groups with available data.analyses were excluded. Source data are provided as a Source Data file. Source data are provided as a Source Data file.

	CCNE1		*NF1*		*PIK3CA*		*RAD21*		*MYC*	
Contrasting groups	**P-value**	**Padj**	**P-value**	**Padj**	**P-value**	**Padj**	**P-value**	**Padj**	**P-value**	**Padj**
BRCA1 LTS vs BRCA1 STS	0.585	0.798	0.651	0.651	0.281	0.52	0.08	0.213	0.322	0.483
BRCA1 LTS vs BRCA2 LTS	0.552	0.798	0.009	0.445	0.561	0.701	0.085	0.213	0.023	0.116
BRCA1 LTS vs BRCA-P LTS	1	1	0.16	0.296	0.312	0.52	0.395	0.539	0.592	0.74
BRCA1 LTS vs BRCA2 STS	0.017	0.051	0.188	0.296	0.281	0.52	0.48	0.6	0.726	0.819
BRCA1 LTS vs BRCA-P STS	<0.001	<0.001	0.445	0.447	0.033	0.233	0.002	0.02	0.012	0.116
BRCA1 STS vs BRCA2 LTS	1	1	0.005	0.045	0.139	0.347	1	1	0.2	0.375
BRCA1 STS vs BRCA2 STS	1	1	0.085	0.255	0.005	0.073	0.078	0.213	0.259	0.432
BRCA1 STS vs BRCA-P LTS	0.099	0.213	0.008	0.045	0.047	0.233	0.698	0.806	0.764	0.819
BRCA1 STS vs BRCA-P STS	<0.001	0.002	0.194	0.296	0.163	0.35	0.201	0.333	0.115	0.288
BRCA2 LTS vs BRCA2 STS	1	1	0.34	0.095	0.126	0.347	0.126	0.235	0.031	0.118
BRCA2 LTS vs BRCA-P LTS	0.225	0.375	0.025	0.095	0.476	0.65	0.848	0.909	0.151	0.324
BRCA2 LTS vs BRCA-P STS	0.002	0.01	0.112	0.281	0.92	0.92	0.103	0.22	1	1
BRCA2 STS vs BRCA-P LTS	0.147	0.276	0.197	0.296	0.423	0.635	0.222	0.333	0.447	0.61
BRCA2 STS vs BRCA-P STS	0.006	0.023	0.319	0.392	0.126	0.347	0.003	0.02	0.019	0.116
BRCA-P LTS vs BRCA-P STS	0.081	0.202	0.225	0.307	0.713	0.764	0.072	0.213	0.076	0.2228

BRCA-P=BRCA-Proficient, STS=Short term survivor, LTS=Long term survivor, Padj=P value adjusted

2.7. Supplementary Table 7

Frequency of genes identified as significant in both differential methylation and differential expression analyses in the multi-omics cohort (n=154). This table summarizes the number of genes identified as significant in both differential methylation (DM) and differential expression (DE) analyses across the indicated contrasting groups. Genes are classified as downregulated and hypermethylated or upregulated and hypomethylated based on concordant changes in gene expression and DNA methylation. Differential methylation and differential expression analyses were performed using two-sided statistical tests. To be included, genes were required to exhibit an absolute delta beta (ADB) > 0.2, indicating a substantial difference in mean DNA methylation beta values between comparison groups. Only genes with Benjamini–Hochberg (BH) adjusted P values (Padj) ≤ 0.1 in both the DM and DE analyses were retained; genes with probes showing Padj > 0.1 in either analysis were excluded.

Contrasting groups	Down regulated and hypermethylated genes	Upregulated and hypomethylated genes
BRCA1 LTS vs BRCA1 STS	0	0
BRCA2 LTS vs BRCA2 STS	0	0
BRCA LTS vs BRCA STS	0	0
BRCA-P LTS vs BRCA-P STS	0	0
BRCA-P LTS vs BRCA1 STS	11	88
BRCA-P LTS vs BRCA2 STS	1	7
BRCA1 LTS vs BRCA-P STS	32	18
BRCA2 LTS vs BRCA-P STS	0	0

BRCA-P=BRCA-Proficient, STS=Short term survivor, LTS=Long term survivor

2.8. Supplementary Table 8

Quartile odds ratios of immune cell subsets comparing short-term versus long-term survival in the multi-omics cohort. This table reports odds ratios (ORs) comparing the relative abundance of immune cell subsets between patients with short-term survival (STS; *n* = 36) and long-term survival (LTS; *n* = 106), stratified by tissue compartment (stromal vs epithelial) and immune cell phenotype. ORs were estimated using logistic regression models comparing STS versus LTS groups, treating immune cell marker expression as quartiles. Reported ORs correspond to the effect per increasing quartile of immune cell density. Two-sided Wald tests were used to assess statistical significance. The 2.5% and 97.5% confidence interval (CI) bounds represent the two-sided 95% confidence interval for each odds ratio. Nominal P-values are shown; no adjustment for multiple comparisons was applied. Source data are provided as a Source Data file.

Immune marker	Area	Cell type	OR	2.5%CI	97.5%CI	*P*-value
CD3+ CD8+	Stromal	T cells CD8+	0.89	0.63	1.24	0.506
CD3+ CD8+	Epithelial	T cells CD8+	0.79	0.55	1.1	0.178
CD3+ CD8+ PD1+	Stromal	T cells CD8+ PD1+	0.69	0.48	0.98	0.043
CD3+ CD8+ PD1+	Epithelial	T cells CD8+ PD1+	0.67	0.46	0.95	0.029
CD3+ CD8-	Stromal	T cells CD4+	0.81	0.57	1.14	0.239
CD3+ CD8-	Epithelial	T cells CD4+	1	0.71	1.4	0.982
CD3+ CD8- PD1+	Stromal	T cells CD4+PD1+	0.89	0.63	1.24	0.506
CD3+ CD8- PD1+	Epithelial	T cells CD4+ PD1+	0.76	0.53	1.07	0.13
CD20+ CD79+	Stromal	B cells	0.97	0.69	1.36	0.88
CD20+ CD79+	Epithelial	B cells	1	0.71	1.4	0.982
CD20- CD79+	Stromal	Plasma cells	0.91	0.65	1.28	0.621
CD20- CD79+	Epithelial	Plasma cells	0.97	0.69	1.36	0.88
CD3+ FOXP3+	Stromal	T-cells regulatory	0.91	0.65	1.28	0.621
CD3+ FOXP3+	Epithelial	T-cells regulatory	0.86	0.61	1.21	0.403
CD68+/PDL1-	Stromal	Macrophages	0.91	0.65	1.28	0.621
CD68+/PDL1-	Epithelial	Macrophages	0.91	0.65	1.28	0.621
CD68+/PDL1+	Stromal	Macrophages PDL1+	0.97	0.69	1.36	0.88
CD68+/PDL1+	Epithelial	Macrophages PDL1+	0.94	0.67	1.32	0.747

OR=Odds ratio, CI=Confidence Interval

3. Supplementary Notes

3.1. Definition of survival groups

The short overall survival (OS) cut-off of <3 years from diagnosis represented the lowest quartile of OS among germline pathogenic *BRCA* variant (g*BRCA*pv) carriers (n=281) in the AOCS cohort (Supplementary Data 1, Supplementary Fig. S1, Supplementary Notes Fig. 1a). When restricted to only *BRCA* carriers who had died (n=170), three years remained a consistent cutoff to identify those in the lowest quartile of OS (Supplementary Notes Fig. 1b).

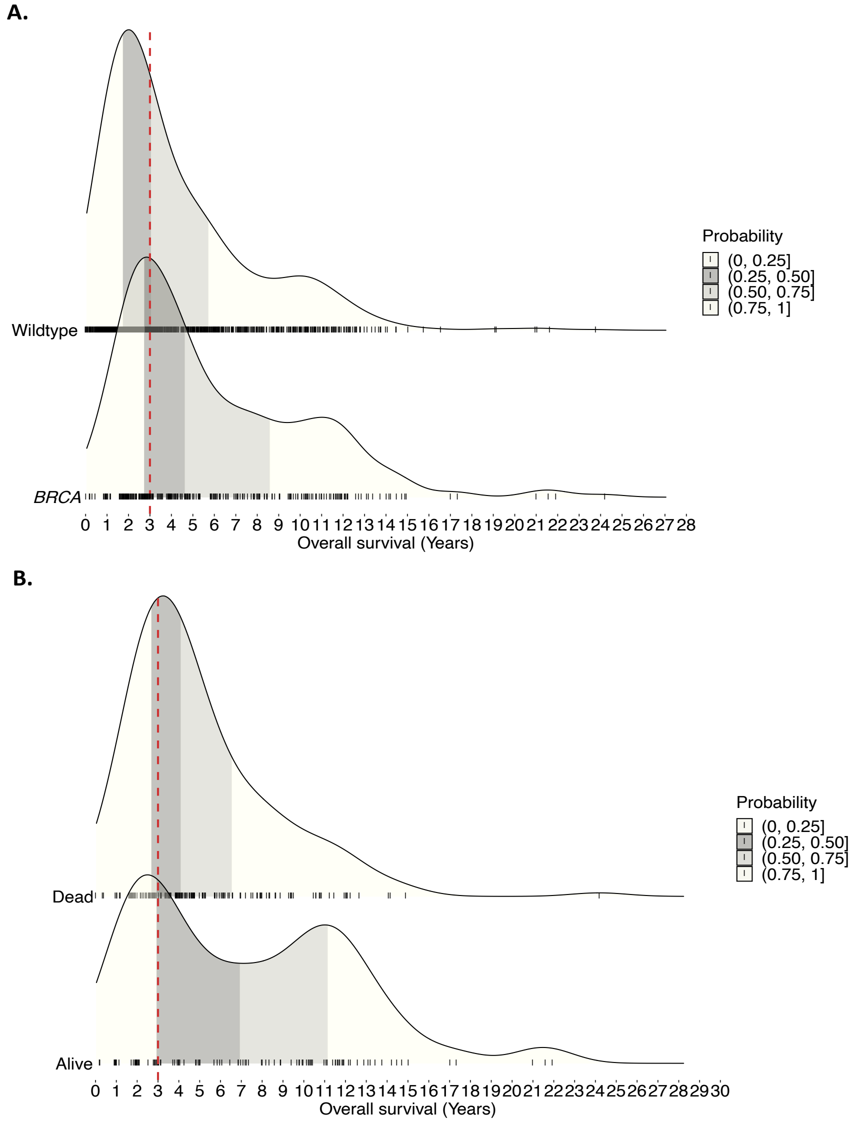

Supplementary Fig. 11 | Survival distribution by *BRCA* status. A. Density plots of overall survival for non carriers (n= 1096 *BRCA* wildtype; top) and patients with germline pathogenic *BRCA* variants (n=281 g*BRCA*pv; bottom), among patients with HGSC and known germline *BRCA* status in the AOCS cohort. **B.** Density plots of overall survival for deceased patients (n=170; top) and those alive at last follow-up (n=111; bottom), all of whom were diagnosed with HGSC and had g*BRCA*pv. The shaded areas represent different quartiles: 0.25-0.50 (ivory), 0.50-0.75 (gray), and 0.75-1.0 (light gray). The red dashed line marks the cut off of three years. Source data are provided as a Source Data file.

3.2. Identification of *BRCA1* and *BRCA2* founder mutations

To determine whether any germline *BRCA1* or *BRCA2* variants detected in the AOCS cohort represented known population founder mutations, we performed a structured, literature-based annotation workflow. First, we compiled a reference list of established founder g*BRCA*pv using authoritative peer-reviewed sources, including comprehensive reviews of global and population-specific founder mutations, and large international *BRCA1/2* variant catalogues from the Consortium of Investigators of Modifiers of *BRCA1/2* (CIMBA) and related analyses [1-6]. These resources provide validated founder alleles across Ashkenazi Jewish, Icelandic, Dutch/Northern European, Polish/Eastern European, Russian/Slavic, Spanish/Iberian, and French-Canadian populations.

All g*BRCA*pv observed in our cohort were then cross-referenced against this curated founder set. Variants were additionally checked in ClinVar and ClinVar Miner for explicit founder annotations and population associations to verify founder status. Only variants consistently described as founder alleles in at least one peer-reviewed population genetics study or a curated clinical database were classified as founder mutations. Variants lacking such evidence, including recurrent or commonly pathogenic mutations, were classified as non-founder.

Founder status and corresponging population were added to Supplementary Data 2 for each g*BRCA*pv in the AOCS cohort.

3.3. BRCA-deficiency

To classify tumors as *BRCA*-deficient, several of levels of evidence were considered: i) germline and tumor samples were evaluated for pathogenic or likely pathogenic alterations in the ovarian cancer risk and/or homologous recombination (HR) DNA repair genes *BRCA1* and *BRCA2* including promoter methylation of *BRCA1*, ii) pathogenic or likely pathogenic alterations in additional HR genes (*RAD51C*, *RAD51D*, *PALB2*, *FANCD2*, and *BRIP1*) were also evaluated, iii) the extent of genomic scarring associated with HR deficiency was assessed in tumor DNA using CHORD (CHORD score > 0.1). For analyses that required classifying samples as either *BRCA1* or *BRCA2* deficient, if there were multiple HR gene alterations in a tumor sample, the more dominant CHORD *BRCA1*- or *BRCA2*-type classification was selected. Tumor samples without an

HR gene alteration and a CHORD score < 0.1 were classified as *BRCA*-proficient. As an additional validation step, we applied HRDetect [7] to tumor genomes and compared these predictions with our manual and CHORD-based classifications. Using the HRDetect cutoff of >0.7 [8], 100% of *BRCA1*-deficient tumors (71/71) and 94% of *BRCA2*-deficient tumors (31/33) were classified as HR deficient, whereas only 6% of *BRCA*-proficient tumors (3/49) exceeded this threshold (Supplementary Data 5). These results demonstrated strong concordance and support the robustness of our *BRCA*-deficiency curation approach.

3.4. Sample quality control

Median tumor cell purity estimated by FACETS [9] was 0.63, with values ranging from 0.30 to 0.92 (Supplementary Data 14). BAMixChecker [10] was run on the paired Tumor/Normal WGS and RNAseq samples to look for sample concordance. One RNAseq sample "BRCA_5" was excluded because of its low concordance rate with the WGS data from the paired tumor sample.

3.5. Mutational signatures

We evaluated all mutational signatures from COSMIC (Catalogue Of Somatic Mutations In Cancer), SIGNAL, and Pan-cancer compendium. We then analyzed these mutational signatures in our cohort as previously described [11].

Signature fitting was conducted in two steps using the R package `signature.tools.lib` and its function `SignatureFit_withBootstrap`, following the methodology described previously [11]. Briefly, an initial fitting was performed using single-base substitutions (SBSs), double-base substitutions (DBSs), small insertions and deletions (IDs), copy number (CN) variations, ovarian cancer specific structural variants (SV) signatures, and chromosomal instability signatures which generated absolute signature exposures or contributions per sample, per signature. A second fitting focused on just ovary-specific signatures from COSMIC (for SBS, DBS, ID, and CN), SIGNAL (for SV), and the Pan-cancer compendium (chromosomal instabilities). Additional signatures present at high levels in the cohort were also included. Signatures were selected based on visualizing the mean signature exposures across the cohort and selecting an acceptable threshold. Absolute

contributions were then converted into relative contributions by dividing each absolute value by the sample's total.

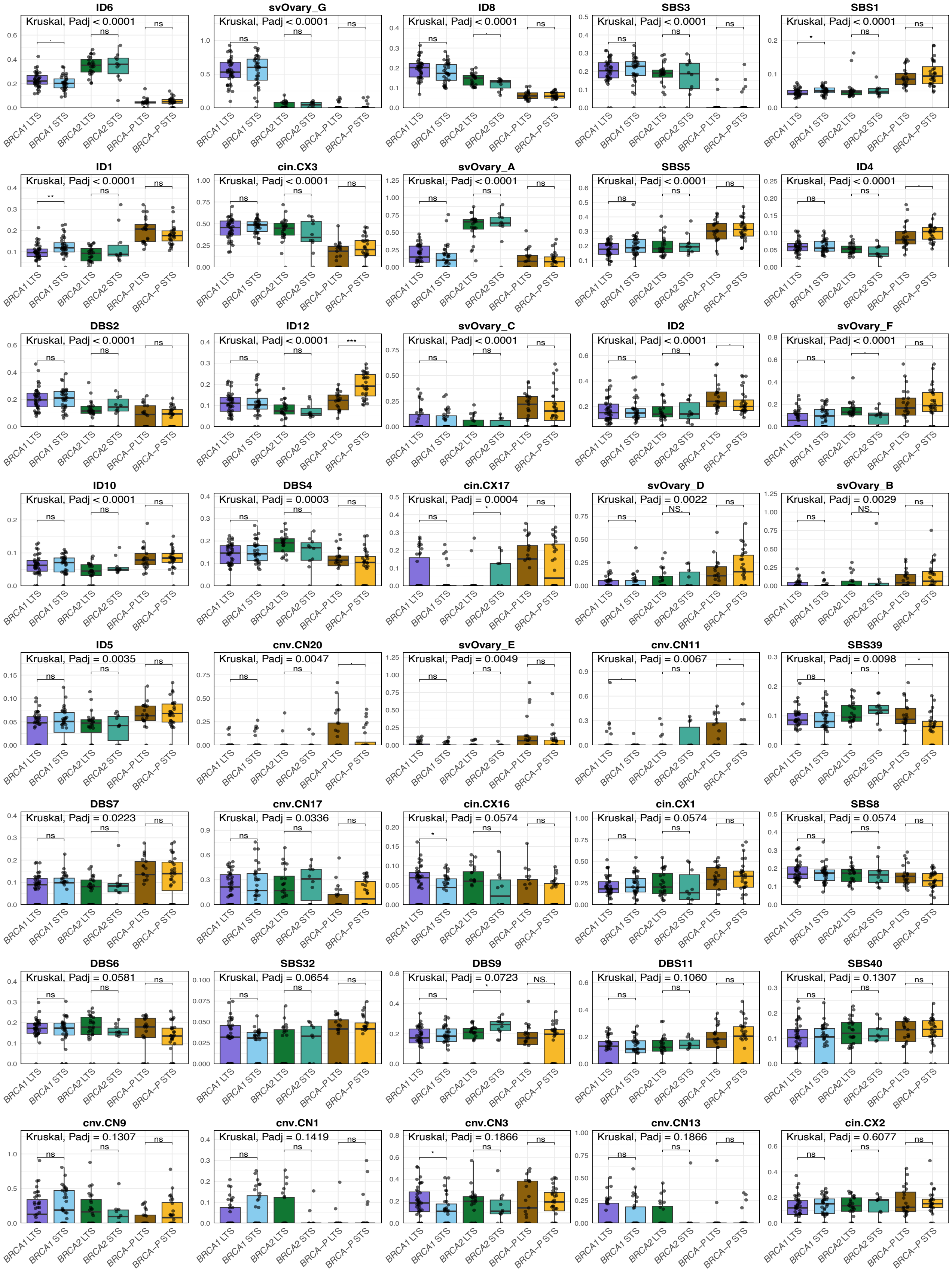

Supplementary Fig. 12 | Distribution analysis of mutational signatures by *BRCA* and survival groups in HGSC. A. Box plots summarizing mutational signatures by *BRCA* and survival groups (n = 154 patients); dots represent each sample, boxes show the interquartile range (25-75th percentiles), central lines show the median, whiskers show the smallest/largest values within 1.5 times the interquartile range. Kruskal-Wallis test Benjamini-Hochberg adjusted *P*-values and pairwise Mann-Whitney-Wilcoxon test P values (two sided) are reported red (ns, *P*>0.1; ·, *P*<0.1; *, *P* <

0.05; **, $P < 0.01$; ***, $P < 0.001$). Features are ranked according to their significance. Source data are provided as a Source Data file.

3.6. NF1 immunohistochemistry (IHC) analysis

Tumor samples were analyzed for NF1 protein expression using IHC. The samples were classified into distinct categories: NF1 inactivated, where NF1 expression was absent in tumor cells but preserved in internal control tissues (e.g., stromal cells); NF1 retained, where normal NF1 protein expression was observed in the tumor cells; subclonal NF1 loss, indicating partial or patchy loss of NF1 expression in some but not all tumor regions; uninterpretable, where NF1 expression was absent in tumor cells but no internal control was available to validate the result; and excluded, where no viable tumor tissue was present for assessment (Supplementary Notes Fig. 3). Following this classification, survival analysis was conducted to compare OS in patients with HGSC based on NF1 expression status (NF1 retained vs. NF1 loss). The analysis was also stratified by germline *BRCA* mutation status, allowing for a more refined evaluation of the interaction between NF1 status and *BRCA* mutations in determining patient outcomes. Results from this analysis are presented in Supplementary Fig. 7a,b.

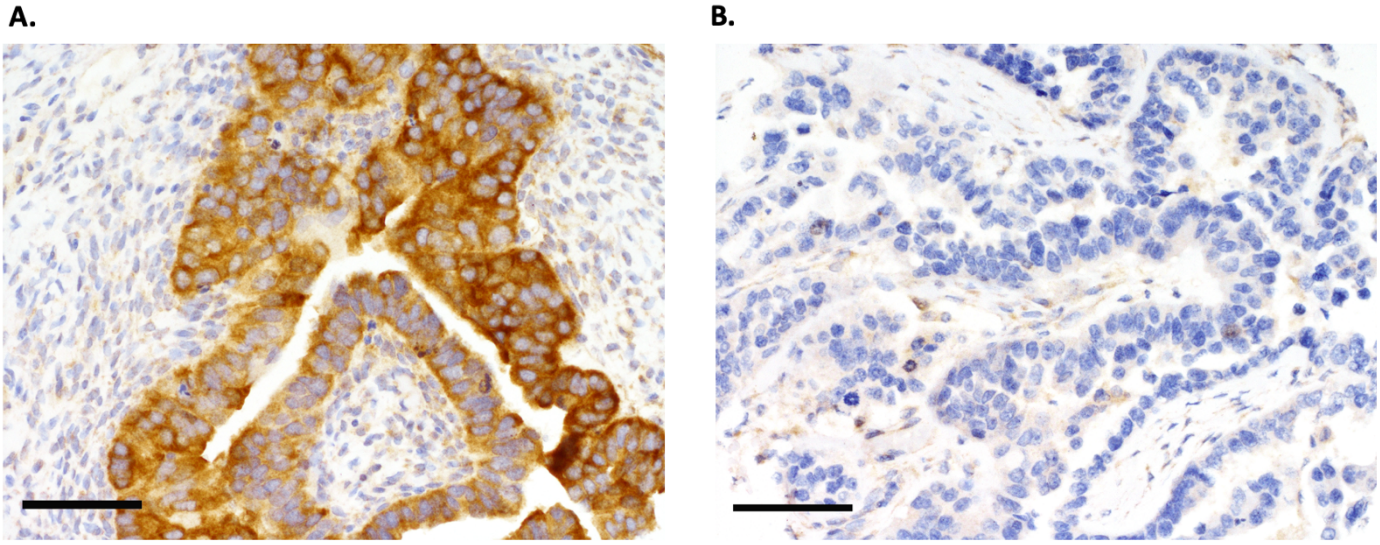

Supplementary Fig. 13 | Representative immunohistochemistry images showing NF1 protein expression patterns in HGSC tumor samples (n = 895 patients). a, illustrates retained NF1 expression, with strong and uniform cytoplasmic staining observed in the tumor cells, indicating normal NF1 protein presence. The surrounding stromal tissue serves as an internal control with weaker staining. **b,** demonstrates complete loss of NF1 protein expression in the tumor cells, characterized by the absence of cytoplasmic staining, while stromal cells retain weak NF1 expression, serving as a reliable internal control for interpretation. These patterns were used to classify tumors as either NF1 retained or NF1 lost for further survival analysis. Scale bar, 60 µm.

3.7. Molecular subtype prediction

DeepCC (v0.1.1) [12] was used to classify the molecular subtypes of the tumor samples, using the Tothill dataset for training as previously described [11]. Briefly, to generate the training data, 1000 iterations were run to generate 1000 different models. This was done because each training run gave slightly different results when applied to the same test data. Molecular subtype predictions were then made by applying these models to the functional spectra of our batch-corrected RNA-seq data. The final molecular subtype was selected as the one that was predicted most frequently across the 1000 models.

3.8. Clonality analysis

Clonality estimation was performed as previously described [11]. Briefly, to obtain an estimate of the depth of the DNA repair pathway alterations and the number of variant reads, the alterations were manually checked in IGV. The total copy number at each variant locus was then determined by intersecting the alteration positions with the FACETS copy number data. The clonal or subclonal status of each variant was then classified by purity and copy number corrected cancer cell fraction (CCF). The CCF was calculated using the "absolute.cancer.cell.fraction" function [freely available at https://github.com/ucl-respiratory/preinvasive], by providing the total depth, variant read counts and FACETS total copy number for each alteration along with FACETS purity estimates for each sample.

3.9. Mutual exclusivity

Pairwise Fisher's Exact tests were conducted to assess the mutual exclusivity or co-occurrence of changes in genes of interest using the "somaticInteractions" function in maftools (v2.2.10) [11]. Only genes altered in at least three primary cancers were included in the analysis. The resulting *P*-values were adjusted for multiple hypothesis testing using the Benjamini-Hochberg method.

3.10. *MYC* amplification, RNA expression, and outcome in *BRCA2*-deficient HGSC

MYC amplification was frequent in this cohort, occurring in 28% of tumors (43/154) (Fig. 3a; Supplementary Data 13). *MYC* amplification was enriched in *BRCA2*-deficient tumors from patients with short compared to long overall survival (5/10, 50% vs 3/24, 12.5%; *P*adj = 0.126), alongside *PIK3CA* and *RAD21* amplifications (Fig. 3a,d,e; Supplementary Fig. S4a).

Mutual exclusivity analysis demonstrated a significant co-occurrence of *RAD21* and *MYC* alterations (23/28 events; *P*adj < 0.001; Supplementary Data 9). Co-amplification of *RAD21* with *MYC* was observed in 20.6% (7/34) of *BRCA2*-deficient tumors (Supplementary Data 9 and 10).

Across *MYC*-amplified tumors, segment-level copy numbers ranged from 7 to 42 copies, with corresponding log R ratios between 1.05 and 3.41, consistent with whole-genome amplifications (Supplementary Data 13). Despite these high-level copy number gains, *MYC* RNA expression showed only a weak correlation with copy number (R=0.21, *P*=0.011). Furthermore, *MYC*-amplified tumors (≥7 copies) did not exhibit significantly higher *MYC* RNA expression compared to non-amplified tumors (*P*=0.13; Supplementary Notes Fig. 4b).

Clinically, survival analyses based on genomic alterations indicate that *MYC* amplification contributes to adverse outcome in *BRCA2*-deficient tumors, particularly in the context of co-amplification with *RAD21* (Supplementary Notes Fig. 4c). Analyses based on *MYC* RNA expression reveal more modest survival differences among g*BRCA1*pv and g*BRCA2*pv carriers, with not as strong prognostic separation (Supplementary Notes Fig. 4d). Together, these findings suggest that the prognostic impact of *MYC* in *BRCA*-deficient tumors is primarily detectable at the genomic level, whereas this effect is not consistently reflected at the transcriptomic level. However, in *BRCA*-proficient tumors, *MYC* showed a clearer relationship with outcome, with amplification and high expression being associated with better survival (Supplementary Notes Fig. 4c,d).

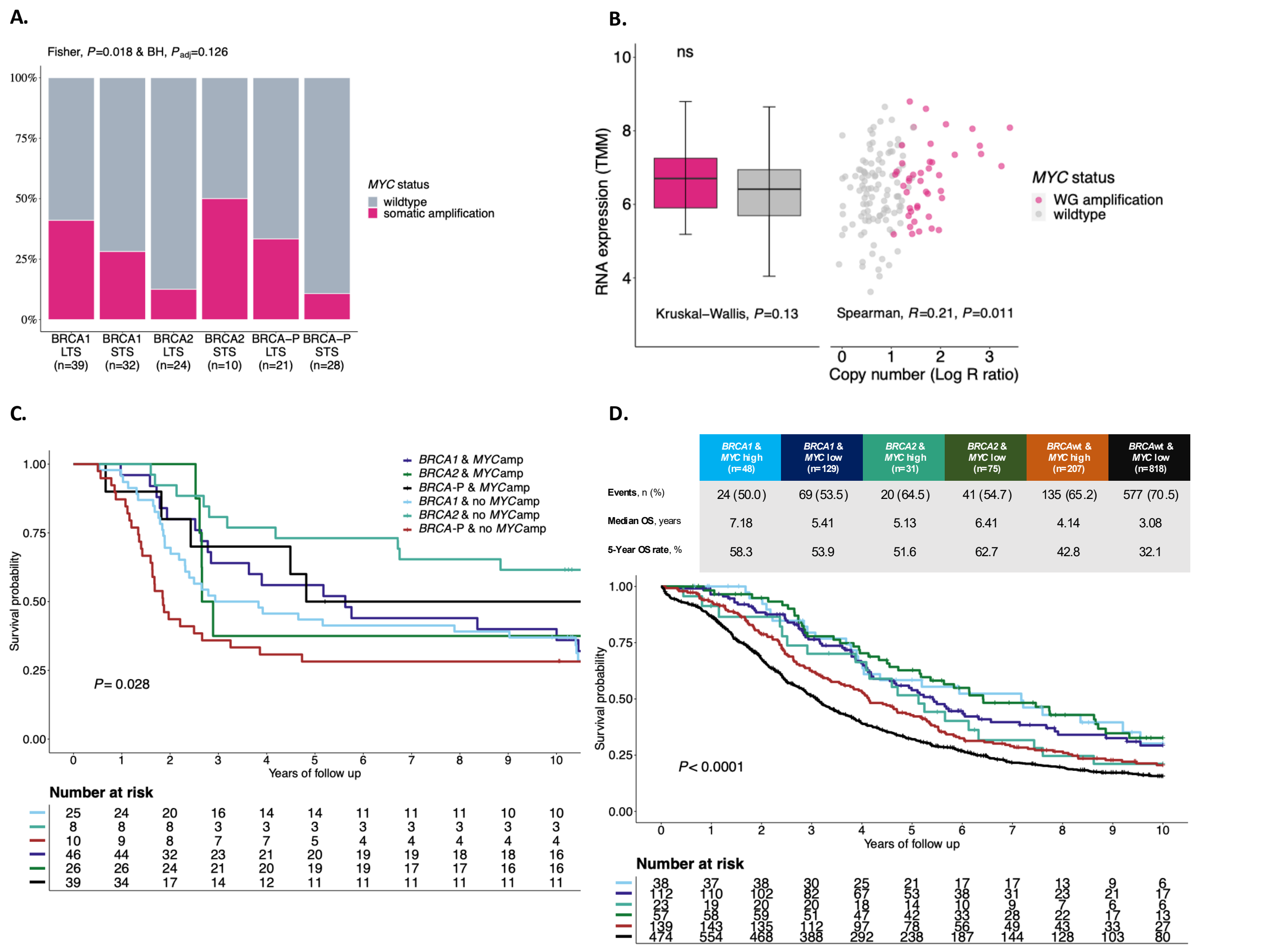

Supplementary Fig. 14 | a, Distribution of *MYC* alteration types across *BRCA* survival groups (n = 154 patients). *P*-values were calculated by the Fisher's exact test and Benjamini-Hochberg (BH) adjusted method (P_{adj}). **b,** Scatter graph (right) of the expression (y-axis) of *MYC* plotted against copy number (x-axis) in primary tumors (n=153, Spearman correlation analysis). Boxplots (left) summarize expression by mutation type; lines indicate median, and whiskers show range. Kruskal–Wallis test *P* value is reported as well as pairwise Wilcoxon rank-sum test *P* values comparing altered groups to wildtype (non-significant (ns), P>0.05; ****, P <0.0001; ***, P <0.001; **, P <0.01). **c**, Kaplan-Meier analysis of overall survival in patients (n=154) with HGSC stratified by *BRCA*-status and *MYC* amplification vs no amplification. *P* value was calculated by log-rank test. **d**, Kaplan-Meier analysis of overall survival in patients (n=1308) with HGSC from the OTTA cohort stratified by *PIK3CA* RNA expression status (high=highest quantile, low=1st to 4th quantiles) and stratified by germline *BRCA* mutation status. *P* value calculated by log-rank test. Source data are provided as a Source Data file.

BRCA status group: Long-term survivor (LTS) = OS >3 years, Short-term survivor (STS) = OS ≤3 years, BRCA-P=BRCA-proficient, WG=Whole gene, BH=Benjamini-Hochberg

3.11. CIBERSORTx analysis

Immune Cell Deconvolution: We employed CIBERSORTx [13], a web-based tool available at https://cibersortx.stanford.edu/, for estimating immune cell populations within the tumor microenvironment. The analysis was configured with batch correction enabled, using absolute mode and 500 permutations, while quantile normalization was disabled. Deconvolution was based on the LM22 signature matrix.

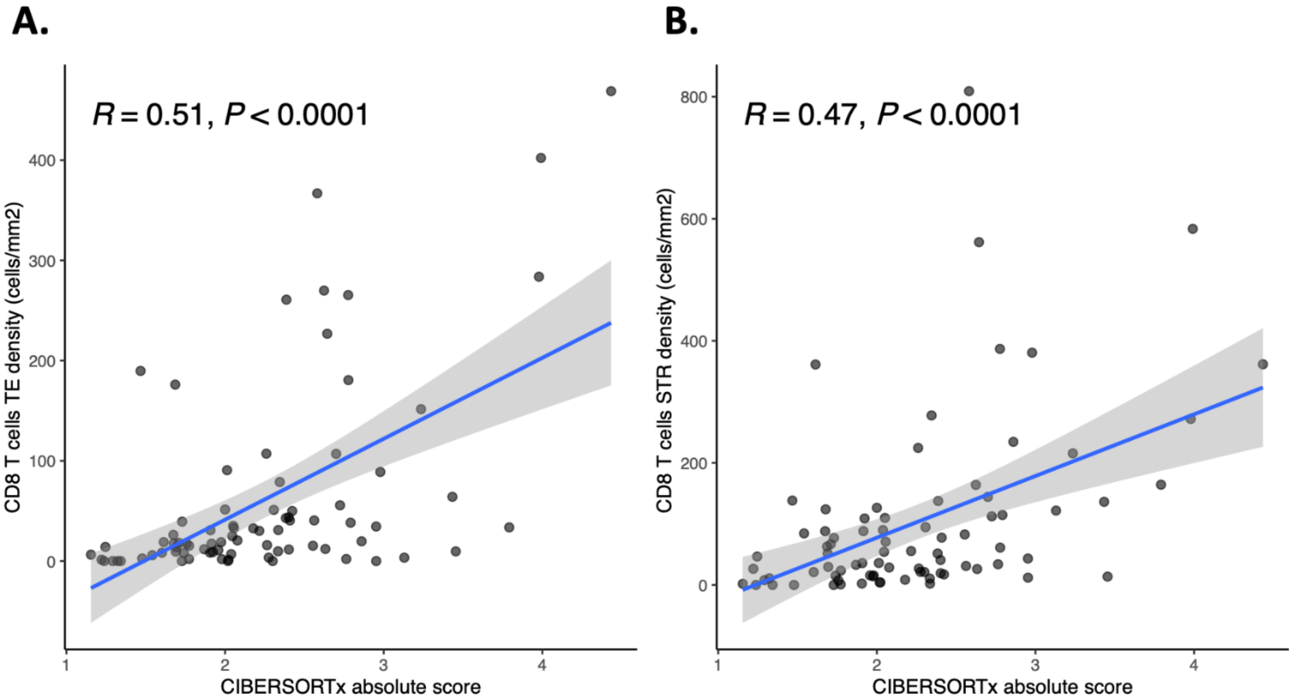

Supplementary Fig. 15 | Comparison of RNA-seq-based immune cell estimation with immunofluorescence staining. The correlation between CIBERSORTx absolute scores and CD8 T-cell densities was used to compare RNA-seq-based immune cell estimation with immunoflurescence staining (n = 81 patients). Scatter plots showing the relationship between CIBERSORTx absolute scores and CD8 T cell densities (cells/mm^2) for **a,** tumor epithelium (TE) and **b,** stroma (STR). In both plots, the blue line represents the linear regression fit and the shaded area indicates the 95% confidence interval. Spearman correlation *P* values are reported (two-tailed).

CIBERSORTx and Immunofluorescence Dataset Comparison: The Spearman correlation test was employed to explore the relationship between CIBERSORTx results and immunofluorescence data. Specifically, we assessed the correlation between CD8+ T cell counts derived from the immunofluorescence data and the immune cell type specific absolute scores obtained from CIBERSORTx (Supplementary Notes Fig. 5). This analysis aimed to evaluate the consistency and reliability of CD8+ T cell quantification across both methods.

Immune Cell Clustering: Using the absolute cell abundance data from CIBERSORTx, we performed clustering using ConsensusClusterPlus [14].

1. Selection of Immune Cell Types: Only cell types with a nonzero value in at least 10 samples were included (Supplementary Notes Fig. 6).
2. Cluster Generation: After selecting the cell types as per the criteria above, the sample by cell type matrix of absolute enrichment scores we used to generate the immune cell clusters following a methodology similar to the mutational signature cluster analysis.

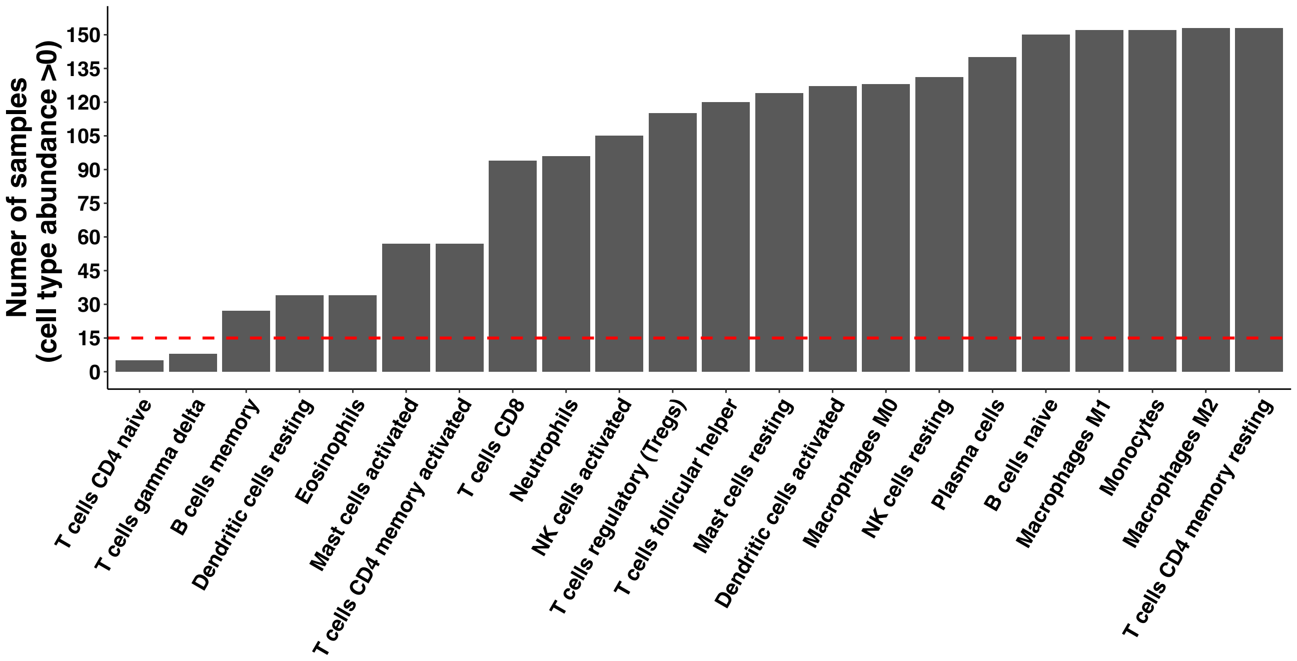

Supplementary Fig. 16 | Cell type selection for use in clustering analysis. Bar chart showing the distribution of cell type abundances from CIBERSORTx analysis (n = 153 patients). The x-axis lists all immune cell types. The y-axis represents the number of samples in which each cell type is detected with an abundance greater than zero. This visualization highlights the prevalence of different immune cell types across the analyzed samples. The cut-off for cell types included in the clustering is indicated by the red dashed line. Source data are provided as a Source Data file.

3.12. Methylation analyses

Methylation classification of gene promoters: To determine the methylation status of the *BRCA1* and *RAD51C* gene promoters, we analyzed their mRNA expression levels in relation to the methylation array beta values of probes situated in the 5' CpG island of each gene. This analysis was conducted across all primary tumor samples (n = 153) using previously established methods [11]. To determine the methylation status of the *BRCA1* and *RAD51C* gene promoters, we analyzed their mRNA expression levels with respect to to the methylation array beta values of probes located in the 5' CpG island of each gene. This analysis was performed on all primary tumor samples (n = 154) using previously established methods. Specifically, we selected methylation probes that showed a significant negative correlation with mRNA expression (Spearman's r < -0.30, P < 0.01). For *BRCA1*, 15 probes (cg04110421, cg04658354, cg08386886, cg09441966, cg09831010, cg10893007, cg15419295, cg16630982, cg16963062, cg18372208cg, 19088651, cg19531713, cg20187250, cg21253966, and cg24806953), and *RAD51C*, 2 probes (cg02118635 and cg24099023) were selected. Samples were classified as methylated if their beta values exceeded 0.2 in at least 50% of the selected probes

and/or the samples were classified as methylated as previously analyzed with the same method on the 126 primary tumors included in the previous study [11].

Differential Methylation vs Differential Expression analysis

Differential Methylation (DM): A combined beta matrix of 154 patients and 364,185 probes was generated by intersecting the common probes between the 450k and EPIC methylation assays. The beta values were converted to M-values [15] and the R package Limma (v3.48.2) was used to identify differentially methylated probes between the groups of interest. A batch effect was observed using multidimensional scaling (MDS) plots between the methylation assays and was subsequently included in the model during the DM analysis.

Differential Expression (DE): Please refer to section "*Differential expression analysis*" in the main methods.

We used starburst plots to assess the interaction between methylation and expression [16].

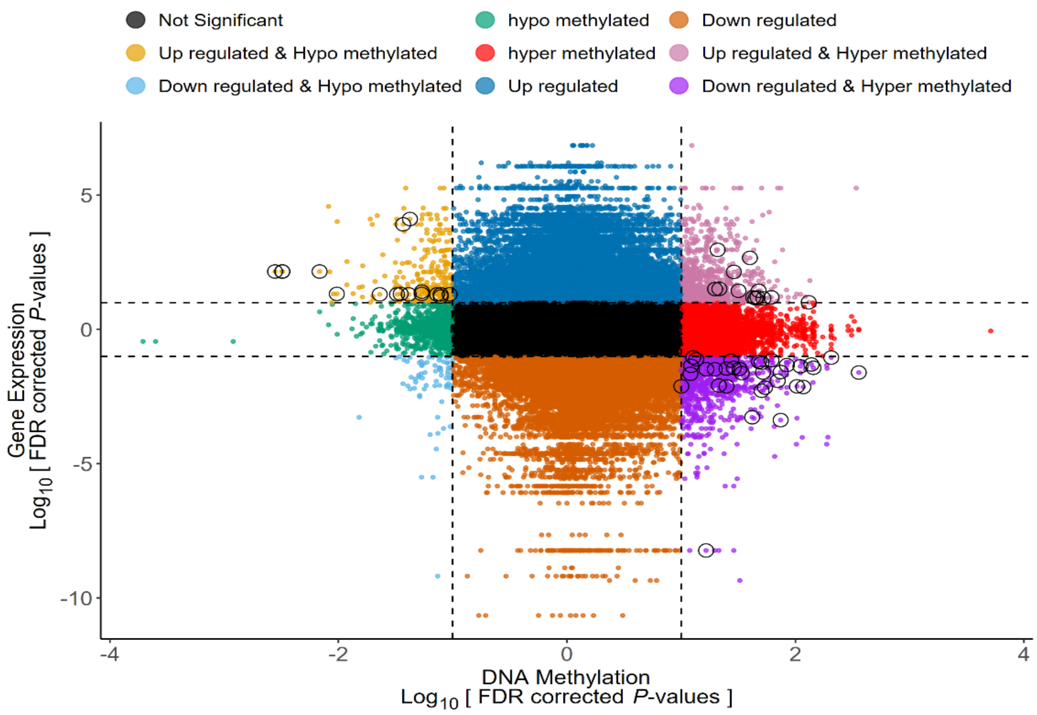

Supplementary Fig. 17 | Starburst plot integrating differential DNA methylation and gene expression. Starburst plots show the relationship between differential DNA methylation (DM) and differential gene expression (DE). DM analysis was performed on 364,185 probes across 154 samples shared between the Illumina 450K and EPIC methylation arrays. Signed log10 adjusted *P*-values (P_{adj}) from DM (x-axis) and DE (y-axis) analyses are shown, with dotted lines indicating the significance threshold ($P_{adj} < 0.1$). Colored dots represent combinations of methylation and expression changes, while black dots indicate non-significant probes/genes. Circled points highlight probes significant in both analyses with an absolute delta beta (ADB) > 0.2. *FDR, False discovery rate*

To generate data for the starburst plots, the P_{adj} values from the DM and DE analyses were converted to $-\log_{10}$ (P_{adj}) values and the direction of the fold changed was applied to these values (signed P_{adj} values). The signed P_{adj} values were then plotted along the X-axis for methylation, and Y-axis for expression. The dotted lines

indicate a P_{adj} value of < 0.1 and the colored dots indicate various groups of interaction between the methylation and expression data. The black dots in the center denote genes and probes with no significant interaction in the DM vs DE analysis (annotated "Not Significant" in the legend). The circled dots are probes that are significant in both the DM and DE analysis with an ADB (Absolute Delta Beta) of > 0.2 to indicate a notable difference in the mean beta values between the contrasting groups. Only significant probes with P_{adj} < 0.1 for both DM and DE analyses that fell in the top-left (Up regulated & Hypo methylated), top-right (Up regulated & Hyper methylated), bottom left (Down regulated & Hypo methylated) and bottom-right (Down regulated & Hyper methylated) were output. We then focused on the bottom right and top left portion of the starburst plot i.e. the group of genes that were significantly (P_{adj} < 0.1) "Down regulated & Hyper methylated" or "Upregulated & Hypo methylated" with ADB > 0.2 (Supplementary Table 8).

3.13. D11q analysis

Creation of d11q Expression Data: Reads mapping to BRCA1 coordinates (GRCh38 17:41196312-41277468) were extracted into individul BAM files for each sample (n=153). The BAM files and a GTF containing the canonical and d11q BRCA1 gene models were then fed into the R library ASpli (v2.0.0) to generate splice junction counts. The d11q splice junction proportions were calculated as [d11q_splice_junction_counts/(d11q_splice_junction_counts + canonical_splice_junction_counts)] where "d11q_splice_junction_counts" were reads mapping to the d11q splice junction and "canonical_splice_junction_counts" were reads mapping to the canonical splice junction.

Selection and Analysis of d11q Expression: The d11q expression data was filtered using a threshold of 10 for d11q exon counts. After applying this threshold, 122 samples remained for the final analysis (Supplementary Data 4**)**. The selected samples were then subjected to further statistical and bioinformatic analyses to explore the expression patterns and their potential implications in the study (Fig. 2b-e**)**.

3.14. Analysis of the locus specific loss of heterozygosity in *NF1* alterations

To assess the locus specific loss of heterozygosity (LOH) in *NF1* alterations, we integrated tumor sequencing data, including variant and reference allele read counts, variant allele frequencies (VAFs), clonality, and lower copy number (LCN) (Supplementary Data 8). Tumor VAFs were calculated as the proportion of variant allele reads to total reads. These observed tumor VAFs were then compared to the expected values based on LCN and clonality. Locus specific LOH was indicated in tumors with reduced copy number (LCN = 0). Discrepancies between observed and expected VAFs were evaluated for potential contributions from tumor purity, technical artifacts, or additional copy number alterations. Locus specific LOH was determined by concordant evidence from elevated tumor VAFs, copy number status, and clonality. This classification was further supported by the depletion of mRNA expression in tumors with NF1 alterations exhibiting locus specific LOH ($P<0.0001$; Supplementary Fig. S6a).

3.15. Analysis of wildtype loss in *BRCA* mutated tumors

To assess the loss of the wildtype allele in *BRCA* cases, we integrated germline and tumor sequencing data, including variant and reference allele read counts, VAFs, clonality, and LCN (Supplementary Data 6). Germline and tumor VAFs were calculated as the proportion of variant allele reads to total reads. Germline VAFs were validated to confirm heterozygosity, typically around 50%.

The tumor VAFs were compared to the expected values based on LCN and clonality. For tumors with normal copy number (LCN = 1), retention of the wild-type allele was indicated by a tumor VAF approximating the germline value, while loss of the wild-type allele (LOH) was inferred from VAFs approaching 100%. For reduced copy number (LCN = 0), tumor VAF near 100% indicated loss of the wild-type allele.

While LCN was used as the primary metric for assessing allelic loss, total copy number (TCN) data provided additional context, with TCN = 1 suggesting loss of a single allele and TCN = 0 indicating homozygous deletion. Discrepancies between observed and expected VAFs were evaluated for potential contributions from tumor purity, technical artifacts, or additional copy number alterations. Loss of the wild-type allele was determined by concordant evidence from elevated tumor VAFs, copy number status, and clonality.

4. **Supplementary References**

1. Kechin, A., et al., *A spectrum of BRCA1 and BRCA2 germline deleterious variants in ovarian cancer in Russia.* Breast Cancer Res Treat, 2023. **197**(2): p. 387-395.
2. Heramb, C., et al., *BRCA1 and BRCA2 mutation spectrum - an update on mutation distribution in a large cancer genetics clinic in Norway.* Hered Cancer Clin Pract, 2018. **16**: p. 3.
3. Laraqui, A., et al., *BRCA genetic screening in Middle Eastern and North African: mutational spectrum and founder BRCA1 mutation (c.798_799delTT) in North African.* Dis Markers, 2015. **2015**: p. 194293.
4. Infante, M., et al., *The highly prevalent BRCA2 mutation c.2808_2811del (3036delACAA) is located in a mutational hotspot and has multiple origins.* Carcinogenesis, 2013. **34**(11): p. 2505-11.
5. Janavicius, R., *Founder BRCA1/2 mutations in the Europe: implications for hereditary breast-ovarian cancer prevention and control.* EPMA J, 2010. **1**(3): p. 397-412.
6. Laitman, Y., et al., *The spectrum of BRCA1 and BRCA2 pathogenic sequence variants in Middle Eastern, North African, and South European countries.* Hum Mutat, 2019. **40**(11): p. e1-e23.
7. Davies, H., et al., *HRDetect is a predictor of BRCA1 and BRCA2 deficiency based on mutational signatures.* Nat Med, 2017. **23**(4): p. 517-525.
8. Nguyen, L., et al., *Pan-cancer landscape of homologous recombination deficiency.* Nat Commun, 2020. **11**(1): p. 5584.
9. Shen, R. and V.E. Seshan, *FACETS: allele-specific copy number and clonal heterogeneity analysis tool for high-throughput DNA sequencing.* Nucleic Acids Res, 2016. **44**(16): p. e131.
10. Chun, H. and S. Kim, *BAMixChecker: an automated checkup tool for matched sample pairs in NGS cohort.* Bioinformatics, 2019. **35**(22): p. 4806-4808.
11. Garsed, D.W., et al., *The genomic and immune landscape of long-term survivors of high-grade serous ovarian cancer.* Nat Genet, 2022. **54**(12): p. 1853-1864.
12. Gao, F., et al., *DeepCC: a novel deep learning-based framework for cancer molecular subtype classification.* Oncogenesis, 2019. **8**(9): p. 44.

13. Newman, A.M., et al., *Determining cell type abundance and expression from bulk tissues with digital cytometry.* Nat Biotechnol, 2019. **37**(7): p. 773-782.

14. Wilkerson, M.D. and D.N. Hayes, *ConsensusClusterPlus: a class discovery tool with confidence assessments and item tracking.* Bioinformatics, 2010. **26**(12): p. 1572-3.

15. Xie, C., et al., *Differential methylation values in differential methylation analysis.* Bioinformatics, 2019. **35**(7): p. 1094-1097.

16. Noushmehr, H., et al., *Identification of a CpG island methylator phenotype that defines a distinct subgroup of glioma.* Cancer Cell, 2010. **17**(5): p. 510-22.
