# Supplementary material for: Clinicopathologic and molecular predictors of survival in BRCA-deficient tubo-ovarian high-grade serous carcinoma
Source: Nat Commun. 2026 Apr 1;17:4789. doi: 10.1038/s41467-026-71134-3 (PMC13219410; doi:10.1038/s41467-026-71134-3)
Supplement: Supplementary file 2 — Description of Additional Supplementary Information [file 41467_2026_71134_MOESM2_ESM.pdf]

Supplementary Data legends

Title: Supplementary Data 1
Description: Clinical data of the Australian Ovarian Cancer Study (AOCS) cohort.

Title: Supplementary Data 2
Description: Mutation type and location gBRCApv-carriers Australian Ovarian Cancer Study (AOCS) cohort. *DBD=DNA Binding Domain, RING=Really Interesting New Gene, RAD51-BD=RAD51 Binding Domain, BRCT=BRCA1 C-Terminal, SV=Structural Variant*

Title: Supplementary Data 3
Description: Clinical data of the multi-omics cohort. *gBRCA=pathogenic germline BRCA variation, sBRCA= pathogenic somatic BRCA variation, methBRCA1=hypermethylated BRCA1, STS=Short term survivor, LTS=Long term survivor*

Title: Supplementary Data 4
Description: Mutation location with d11q proportion and d11q BRCA1 expression in the multi-omics cohort. *HRD=Homologous recombination deficiency, gBRCA=pathogenic germline BRCA variation, sBRCA= pathogenic somatic BRCA variation, methBRCA1=hypermethylated BRCA1*

Title: Supplementary Data 5
Description: Overall survival stratified by molecular and clinical features in the multi-omics cohort. Results of the Kaplan-Meier analysis of overall survival in 154 patients with HGSC from the multi-omics cohort stratified by the main features of interest. Source data are provided as a Source Data file. *OS=Overall survival, LCL=Lower Confidence Limit, UCL=Upper Confidence Limit, HR-deficiency=Homologous recombination deficiency, BRCAwt= BRCA wildtype, amp=amplification, BRCA-P=BRCA-proficient, LOH=Loss of heterozygosity*

Title: Supplementary Data 6
Description: BRCA groups, HRD scores, CHORD scores, whole genome duplication, and molecular signatures multi-omics cohort. *HRD=Homologous recombination deficiency, CHORD=Classifier of Homologous Recombination Deficiency, LOH=Loss of heterozygosity,*

TAI=Telomeric allelic imbalance, LST=Large-scale state transitions, WGD=Whole Genome Duplication, BRCAwt=BRCA wildtype gBRCA=pathogenic germline BRCA variation, sBRCA= pathogenic somatic BRCA variation, methBRCA1=hypermethylated BRCA1, STS=Short term survivor, LTS=Long term survivor, BRCA-P=BRCA-Proficient

Title: Supplementary Data 7
Description: Germline alterations in genes of interest with loss of wildtype allele and clonality in the multi-omics cohort. *LOH=Loss of heterozygosity, LCN= Lower copy number, TCN= Total copy number*

Title: Supplementary Data 8
Description: Somatic alterations in genes of interest in the multi-omics cohort

Title: Supplementary Data 9
Description: NF1 alteration type, segment copy number, clonality, loss of heterozygosityand, and RNA expression in the multi-omics cohort. *SV=Structural variant, TCN=Total copy number, LCN=Lower copy number, LOH=Loss of heterozygosity, TMM=Trimmed Mean of M-values*

Title: Supplementary Data 10
Description: Mutual exclusivity and co-occurrence analysis of the multi-omics cohort. This table summarizes pairwise relationships between altered genes, reporting statistically significant co-occurrence or mutual exclusivity. For each gene pair, the association was tested using a two-sided Fisher's exact test based on the presence/absence of alterations across samples. Odds ratios quantify effect size (odds ratio >1 indicates co-occurrence; odds ratio <1 indicates mutual exclusivity; "Inf" indicates no events in one comparison cell). P values were adjusted for multiple comparisons using the Benjamini–Hochberg (BH) procedure (reported as P-value adjusted). "Event" indicates whether the pair is classified as co-occurring or mutually exclusive; "Pair" lists the two genes evaluated; and "Event ratio" reports the number of samples with alterations in both genes relative to the comparator group as defined in the analysis output. Source data are provided as a Source Data file.

Title: Supplementary Data 11

Description: Mutual exclusivity and co-occurrence analysis short survival BRCA group of the multi-omics cohort. This table reports pairwise patterns of co-occurrence and mutual exclusivity within the short-survival BRCA subgroup. For each gene pair, association was assessed using a two-sided Fisher's exact test based on the presence/absence of alterations across samples. The odds ratio provides the effect size (odds ratio >1 indicates co-occurrence; odds ratio <1 indicates mutual exclusivity; "Inf" indicates zero counts in one contingency table cell). Nominal P values are shown (P-value) and P values adjusted for multiple comparisons using the Benjamini–Hochberg (BH) procedure are reported as P-value adjusted. "Event" indicates whether the relationship is classified as co-occurring or mutually exclusive; "Pair" lists the gene pair; and "Event ratio" reports the number of samples with alterations in both genes relative to the comparator group as defined in the analysis output. Source data are provided as a Source Data file.

Title: Supplementary Data 12
Description: PIK3CA segment copy number, RNA expression, and alteration type in the multi-omics cohort. *WG=Whole gene, SV=Structural variant*

Title: Supplementary Data 13
Description: RAD21 segment copy number, RNA expression, and alteration type in the multi-omics cohort. *WG=Whole gene, SV=Structural variant*

Title: Supplementary Data 14
Description: MYC segment copy number, RNA expression, and alteration type in the multi-omics cohort.

Title: Supplementary Data 15
Description: Mutation and neoantigen burden in the multi-omics cohort. *SV=Structural variant, SNV=Single nucleotide variant, MB=Megabase, MHC=Major Histocompatibility Complex*

Title: Supplementary Data 16
Description: Relative CIBERSORTx abundance of LM22 cell types of the multi-omics cohort with cluster. *RMSE=Root Mean Square Error*

Title: Supplementary Data 17

Description: Details of participating study sites and ethics approvals from the Ovarian Tumor Tissue Association (OTTA) cohort.

Title: Supplementary Data 18

Description: Antibodies and staining conditions used for multiplex immunofluorescence.
