# Supplementary material for: Clinicopathologic and molecular predictors of survival in BRCA-deficient tubo-ovarian high-grade serous carcinoma
Source: Nat Commun. 2026 Apr 1;17:4789. doi: 10.1038/s41467-026-71134-3 (PMC13219410; doi:10.1038/s41467-026-71134-3)
Supplement: Supplementary file 4 — Reporting summary [file 41467_2026_71134_MOESM4_ESM.pdf]

nature portfolio

Corresponding author(s): Dale W. Garsed, Tibor A. Zwimpfer

Last updated by author(s): Mar 9, 2026

Reporting Summary

Nature Portfolio wishes to improve the reproducibility of the work that we publish. This form provides structure for consistency and transparency in reporting. For further information on Nature Portfolio policies, see our Editorial Policies and the Editorial Policy Checklist.

Statistics

For all statistical analyses, confirm that the following items are present in the figure legend, table legend, main text, or Methods section.

n/a	Confirmed	
☐	☒	The exact sample size (*n*) for each experimental group/condition, given as a discrete number and unit of measurement
☒	☐	A statement on whether measurements were taken from distinct samples or whether the same sample was measured repeatedly
☐	☒	The statistical test(s) used AND whether they are one- or two-sided<br>*Only common tests should be described solely by name; describe more complex techniques in the Methods section.*
☐	☒	A description of all covariates tested
☐	☒	A description of any assumptions or corrections, such as tests of normality and adjustment for multiple comparisons
☐	☒	A full description of the statistical parameters including central tendency (e.g. means) or other basic estimates (e.g. regression coefficient) AND variation (e.g. standard deviation) or associated estimates of uncertainty (e.g. confidence intervals)
☐	☒	For null hypothesis testing, the test statistic (e.g. *F*, *t*, *r*) with confidence intervals, effect sizes, degrees of freedom and *P* value noted<br>*Give P values as exact values whenever suitable.*
☒	☐	For Bayesian analysis, information on the choice of priors and Markov chain Monte Carlo settings
☐	☒	For hierarchical and complex designs, identification of the appropriate level for tests and full reporting of outcomes
☐	☒	Estimates of effect sizes (e.g. Cohen's *d*, Pearson's *r*), indicating how they were calculated

Our web collection on statistics for biologists contains articles on many of the points above.

Software and code

Policy information about availability of computer code

Data collection

WGS
Paired-end Whole Genome Sequencing (WGS) at 150bp was performed on HiSeq X Ten System machines.
Software used for WGS processing:
- FASTQC (v0.11.8)
- FastQ Screen (v0.11.4)
- fastq-mcf (v1.05)
- BWA mem (v0.7.17-r1188)
- Picard Tools (v2.17.3)
- GATK BaseRecalibrator &HaplotypeCaller (v4.0.10.1)
- GATK DepthOfCoverage (v3.8-1-0-gf15c1c3ef)
- FACETS (v0.6.1)

RNA-seq
Libraries were generated using Illumina Stranded mRNA Prep and 150 bp paired-end sequencing was performed on Illumina NovaSeq 6000 instruments.
Software used for RNAseq processing:
- FASTQC (v0.11.8)
- FastQ Screen (v0.11.4)
- STAR(v2.6.0b)

- Picard Tools (v2.17.3)

Methylation
Tumor DNA was bisulfite converted with the EZ DNA Methylation kit (Zymo Research) and assayed using the Infinium MethylationEPIC BeadChip arrays according to manufacturer's instructions (Illumina).
Software used for Methylation data processing:
- minfi (v1.32.0)

SNP array
Tumor and matched normal DNA was assayed with the Infinium OmniExpress-24 BeadChip arrays, arrays scanned and data processed using Genotyping module 2.0.3 software in GenomeStudio 2.0.3 to calculate logR ratios and B-allele frequencies according to manufacturer's instructions (Illumina).
Software used for SNParray data processing:
- HYSYS github version b730498 (https://github.com/PapenfussLab/HaveYouSwappedYourSamples)
- qPure v1.1 (https://sourceforge.net/projects/qpure/)
- ASCAT v2.5.2 (https://github.com/VanLoo-lab/ascat)

Data analysis

WGS
- VarDictJava (v1.5.7 with –r=2 –Q=10 –f=0.01)
- Mutect2 (v4.0.11.0 with defaults)
- Strelka2 (v2.9.9 with defaults)
- VarScan2 (v2.4.3 with --min-coverage 7 --min-var-freq 0.05 --min-freq-for-hom 0.75 --p-value 0.99 --somatic-p-value 0.05 --strand-filter 0 & SAMtools v1.9 for mpileup)
- vt (v0.57721)
- GATK ReadBackPhasing (v3.8-1-0-gf15c1c3ef with --phaseQualityThresh 10 --enableMergePhasedSegregatingPolymorphismsToMNP --min_base_quality_score 10 min_mapping_quality_score 10 --maxGenomicDistanceForMNP 2)
- GATK CombineVariants (v3.8-1-0-gf15c1c3ef with -genotypeMergeOptions UNIQUIFY -priority Strelka2, Mutect2, VarScan2, VarDictJava)
- GATK VariantAnnotator (v3.8-1-0-gf15c1c3ef with --reference_window_stop 1000 -A HomopolymerRun -A TandemRepeatAnnotator)
- bam-readcount (v0.8.0 with -w 0 --min-mapping-quality 10 –min-base-quality 10 --max-count 100,000,000)
- Ensembl Variant Effect Predictor (VEP v92.4)
- Manta + BreakPointInspector (v1.5.0)
- GRIDSS (v2.0.1)
- Smoove (v0.2.2)
- SvABA (v134)
- StructuralVariantAnnotation (v1.3.1 with maxgap = 10, ignore.strand = FALSE)
- InteractionSet (v1.14.0)
- rtracklayer (v1.46.0)
- snp-pileup(v1.0 with --pseudo-snps 100 --min-map830 quality 10 --min-base-quality 10 --max-depth 5000 --min-read-counts 15,0)
- cnv_facets (v0.13.0 with --nbhd snp=500 --cval=50 1000 --depth=15 5000)
- CHORD (v2.00)
- csaw (v1.20.0)
- ICAMS v2.0.10.9001
- rstatix (v0.7.0)
- signature.tools.lib (v0.0.0.9000 with SignatureFit_withBootstrap [method = "KLD", nboot = 100, randomSeed = 42, threshold_percent = 2, threshold_p.value = 0.05])
- ConsensusClusterPlus (v1.50.0 with maxK = 10, reps=1000, pItem=0.9, pFeature=0.9, clusterAlg="pam", distance="pearson", innerLinkage="ward.D2", finalLinkage="ward.D2", seed=12345678)
- seriation (v1.3.0 with method="OLO")
- HLA-VBSeq (v11_22_2018)
- Jvarkit samviewwithmate (ec2c236)
- samtools (v1.9)
- deepTools (v3.0.0 with parameters --binSize 10 --minMappingQuality 10 --normalizeUsing CPM --skipNonCoveredRegions --samFlagExclude 1024 --outFileFormat bigwig)
- pVACtools pVACseq (v1.3.5)
- dNdScv (v0.0.1.0 with refdb = "hg19", sm = "192r_3w", max_muts_per_gene_per_sample = Inf, max_coding_muts_per_sample = Inf, use_indel_sites= FALSE)
- GRIN (v1.4)
- GISTIC2 (v2.0.23 with -savegene 1 -maxspace 1000 -ta 0.1 -td 0.1 -rx 0 -cap 3 -broad 0 –twoside 1 -res 0.05 -genegistic 0 -v 10)
- maftools (v2.2.10)
- Mutalyzer (v2.0.35)
- R (v4.1.3)

RNA-seq
- HTSeq (v0.10.0 with mode = "intersection-nonempty")
- edgeR (v3.28.1)
- limma (v3.48.2)
- DeepCC (v0.1.1)
- DESeq2 (v1.26.0)
- fGSEA (v1.15.1)
- CIBERSORTx web version as at 05/21/2020 (https://cibersortx.stanford.edu/)

- ConsensusClusterPlus (v1.50.0)
- Arriba (v1.1.0)
- ASpli (v2.0.0)
- R (v4.1.3)

Methylation
- limma (v3.48.2)
- R (v4.1.3)

Multicolour immunofluorescence
- QuPath (v0.2m2)
- R (v4.1.3)

For manuscripts utilizing custom algorithms or software that are central to the research but not yet described in published literature, software must be made available to editors and reviewers. We strongly encourage code deposition in a community repository (e.g. GitHub). See the Nature Portfolio guidelines for submitting code & software for further information.

Data

Policy information about availability of data

All manuscripts must include a data availability statement. This statement should provide the following information, where applicable:

- Accession codes, unique identifiers, or web links for publicly available datasets
- A description of any restrictions on data availability
- For clinical datasets or third party data, please ensure that the statement adheres to our policy

Short survival BRCA dataset: WGS, RNA-seq and SNP array data from short-term survivors generated as part of the current study have been deposited in the European Genome-phenome Archive (EGA) repository (https://ega-archive.org) under accession code EGAS00001008059. WGS and RNA-seq data are available as raw FASTQ files for each sample type (tumor/normal) and SNP array data are available as raw signal intensity files in text format for each sample type (tumor/normal). Controlled access to patient sequence data can be gained for academic use via the EGA, typically for a period of five years from the date the data transfer agreement is fully executed. Information on how to apply for access is available at the EGA under accession code EGAS00001008059. Responses to data requests will be provided within ten business days. The raw methylation data sets have been submitted to the Gene Expression Omnibus (GEO; https://www.ncbi.nlm.nih.gov/geo/) under accession code GSE292140 with no access restrictions.

ICGC dataset: Previously published WGS and RNA-seq data generated as part of the ICGC Ovarian Cancer project61 are available from the EGA repository as a single bam file for each sample type (tumor/normal), under the accession code EGAD00001000877. Due to the sensitive nature of these patient datasets, access is subject to approval from the ICGC Data Access Compliance Office, an independent body who authorizes controlled access to ICGC sequencing data. ICGC SNP array and methylation data sets have been deposited into GEO under accession code GSE65821, without access restrictions. ICGC gene count level transcriptomic data has been deposited into the GEO under accession code GSE209964.

MOCOG dataset: WGS, RNA-seq and SNP array data from long-term survivors generated as part of the MOCOG study22 have been deposited in the EGA repository under accession code EGAS00001005984. WGS and RNA-seq data are available as raw FASTQ files for each sample type (tumor/normal) and SNP array data are available as raw signal intensity files in text format for each sample type (tumor/normal). Controlled access to patient sequence data can be gained for academic use via the EGA, typically for a period of five years from the date the data transfer agreement is fully executed. Information on how to apply for access is available at the EGA under accession code EGAS00001005984. Responses to data requests will be provided within ten business days. The MOCOG cohort raw methylation data sets have been submitted to the GEO under accession code GSE211687, with no access restrictions.

Uniformly processed somatic variant data from the ICGC, MOCOG, and short survival BRCA cohorts is deposited in Synapse under accession code syn65463502 and processed methylation and expression data from all cohorts has been submitted into the GEO under accession codes GSE292140 and GSE292142, without access restrictions.

OTTA dataset: The data underlying the figures and tables are provided in the Source Data file.

Population frequencies of genetic variants can be accessed via the Genome Aggregation Database (gnomAD) at https://gnomad.broadinstitute.org/. Supporting evidence for pathogenicity of genomic alterations can be accessed via ClinVar (https://www.ncbi.nlm.nih.gov/clinvar/), BRCA Exchange (https://brcaexchange.org/) and the TP53 Database (https://tp53.cancer.gov/). The Ensembl ranked order of severity of variant consequences is available at: https://www.ensembl.org/info/genome/variation/prediction/predicted_data.html. Mutational signature reference databases can be accessed via COSMIC (https://cancer.sanger.ac.uk/signatures/) and Signal (https://signal.mutationalsignatures.com/). The LM22 signature matrix used for immune cell deconvolution can be downloaded here: https://cibersortx.stanford.edu/. MSigDB hallmark gene sets can be accessed here: https://www.gsea-msigdb.org/gsea/msigdb/. Illumina methylation probes that were filtered out due to poor performance (e.g. cross reactive or non-specific probes) can be found here: https://github.com/sirselim/illumina450k_filtering. Germline polymorphic sites for reference and variant allele read counts used in FACETS analysis can be found at https://ftp.ncbi.nih.gov/snp/organisms/human_9606_b151_GRCh37p13/VCF/common_all_20180423.vcf.gz. The GTF used for annotation and RNA-seq counts is available here: https://ftp.ensembl.org/pub/grch37/release-92/.

All other data are available within the article and its Supplementary and Source Data files.

Research involving human participants, their data, or biological material

Policy information about studies with human participants or human data. See also policy information about sex, gender (identity/presentation), and sexual orientation and race, ethnicity and racism.

Reporting on sex and gender	This study focuses on tubo-ovarian high-grade serous carcinoma, a disease of the female reproductive system.
Reporting on race, ethnicity, or other socially relevant groupings	Race, ethnicity or other socially relevant groupings were not used as variables in this study.
Population characteristics	Patients were diagnosed with high-grade serous cancer (ovarian, fallopian tube or primary peritoneal carcinoma) confirmed by histopathology review, and received primary chemotherapy incorporating a platinum-based compound. Population characteristics are provided in Table 1 and Supplementary Tables 1 and 7. All patients are female and were aged 24-87 years.
Recruitment	Patients were recruited to the population-based Australian Ovarian Cancer Study (AOCS), Peter MacCallum Cancer Centre (Melbourne, Australia), the Gynaecological Oncology Biobank (GynBiobank), Westmead Hospital (Sydney, Australia) or the Mayo Clinic (Rochester, USA) under Research Ethics Committee approval for each study. Written informed consent was provided by all patients. Participation was voluntary, with no compensation provided to participants.<br>1. Patients over the age of 18 (and under 80 for AOCS) with invasive epithelial ovarian cancer (or primary peritoneal or fallopian tube cancer) were recruited through major cancer treatment centers across Australia (AOCS and GynBiobank) and in the USA (the Mayo Clinic), generally at the time of initial diagnosis.<br>2. Surgical resection samples: Tissue samples for research were taken from tissue removed during surgery and were excess to diagnostic requirements. Sampling of surgical tissue was undertaken by a trained pathologist to ensure that samples taken for the purposes of research did not affect histopathological assessment. Samples were snap-frozen and stored for future research, with patient consent.<br>3. Surgical, pathology, systemic treatment and clinical outcome data was collected from medical records during regular longitudinal clinical follow-up. Cases were selected for the current study based on pre-specified criteria, including advanced stage disease (FIGO Stage III/IV), histology (HGSC), primary treatment (platinum-based chemotherapy), and availability of biospecimens for analysis (snap frozen tumor tissue and blood). This may represent a potential bias towards patients with tumors of sufficient size, and a high proportion of tumor cells within the research sample, to enable analyses.<br>AOCS recruited patients <80 years old, which could result in an increased proportion of long-term survivors compared to studies without an upper age limit, because the chance of surviving >10 years if diagnosed over 80 would be lower (due to older age), however ages are consistent between survival groups and not likely to impact the results. No other sources of selection bias were identified.
Ethics oversight	The Peter MacCallum Cancer Centre Human Research Ethics Committee (HREC).

Note that full information on the approval of the study protocol must also be provided in the manuscript.

Field-specific reporting

Please select the one below that is the best fit for your research. If you are not sure, read the appropriate sections before making your selection.

[x] Life sciences [ ] Behavioural & social sciences [ ] Ecological, evolutionary & environmental sciences

For a reference copy of the document with all sections, see nature.com/documents/nr-reporting-summary-flat.pdf

Life sciences study design

All studies must disclose on these points even when the disclosure is negative.

Sample size	No sample size calculations were performed. A limited number of cases were available given the rarity of high-grade serous ovarian cancer fresh frozen tumor tissue with detailed clinical follow-up and matched germline samples available.
Data exclusions	Two tumor samples were flagged as outliers with a high somatic mutation rate (>20 mutations/Mb): AOCS-076 due to cross sample contamination and AOCS-166 due to a germline mutation in the mismatch repair gene PMS2. The two samples were therefore excluded from further analyses. The RNAseq data for tumor sample BRCA_5 was also excluded because of its low concordance rate with the WGS data from the paired tumor sample.
Replication	Due to the rarity of frozen tumor tissue and the high cost of genomic assays, WGS, RNA-sequencing, SNP arrays and methylation arrays were performed once for each sample. Supporting evidence for variants detected by WGS were sought in orthogonal datasets, such as SNP array, clinical sequencing, RNA-sequencing or DNA sequencing of matched samples (e.g. germline and tumor) from the same patient. CIBERSORTx immune scores were verified with CD8+ T cell scores determined by multi-color immunohistochemistry and automated image scoring from our previous study (Garsed et al 2018). Mutational signature analyses were informed by and/or consistent with previous analyses of breast, ovarian and pan-cancer genomic datasets (Alexandrov et al 2013, Patch et al 2015, Popova et al 2016, Alexandrov et al 2020, Degasperi et al 2020, and Garsed et al 2022). Where feasible, we validated findings by generating orthogonal data in independent samples (NF1) or sought validation of findings in pre-existing, independent datasets (The Cancer Genome Atlas Research Network et al 2011; Millstein et al 2020; Talhouk et al 2020).
Randomization	Randomization was not applicable as this study did not involve an intervention. Patient groups with different survival outcomes were compared with patients with similar known survival predictors: histology, grade, age at diagnosis, stage, treatment, etc.

Blinding	For in-depth genomic analyses, blinding was not feasible. Pathologists and laboratory technicians carrying out quantification of cells or markers by multicolour immunofluorescence or immunohistochemistry were blinded to outcomes/groups. All molecular and clinical data was uniformly processed and analyzed across all samples/cases without consideration of patient covariates.

Reporting for specific materials, systems and methods

We require information from authors about some types of materials, experimental systems and methods used in many studies. Here, indicate whether each material, system or method listed is relevant to your study. If you are not sure if a list item applies to your research, read the appropriate section before selecting a response.

Materials & experimental systems

n/a	Involved in the study	
☐	☒	Antibodies
☒	☐	Eukaryotic cell lines
☒	☐	Palaeontology and archaeology
☒	☐	Animals and other organisms
☒	☐	Clinical data
☒	☐	Dual use research of concern
☒	☐	Plants

Methods

n/a	Involved in the study	
☒	☐	ChIP-seq
☒	☐	Flow cytometry
☒	☐	MRI-based neuroimaging

Antibodies

Antibodies used

- NF1 antibody, Anti-Neurofibromin C Antibody, clone NFC, #MABE1820, Sigma-Aldrich

Antibodies used for multiplex immunofluorescence
- CD79a antibody, Anti-CD79a Antibody, clone SP18, #ab16698, Abcam
- CD20 antibody, Anti-CD20 Antibody, clone L26, #CM004, Leica Biosystems
- CD8 antibody, Anti-CD8 Antibody, clone C8/144B, #108M-94, Cell Marque
- CD3 antibody, Anti-CD3 Antibody, clone PS1, #CM110, Leica Biosystems
- FOXP3 antibody, Anti-FOXP3 Antibody, clone 236A/E7, #14-4777-82, Thermo Fisher Scientific
- Pan-cytokeratin antibody, Anti-Pan-Cytokeratin Antibody (AE1/AE3 + 5D3), clone AE1/AE3 + 5D3, #CM162, Leica Biosystems
- CD68 antibody, Anti-CD68 Antibody, clone SP251, #ab192847, Abcam
- PD-1 antibody, Anti-PD-1 Antibody, clone EPR4877(2), #ab137132, Abcam
- PD-L1 antibody, Anti-PD-L1 Antibody, clone SP142, #M4422, Spring Bioscience

Multiplex reagents and counterstain
- Opal fluorophore panel, Opal™ Multiplex IHC Reagent Pack, #FP1501001KT, Akoya Biosciences
- Opal 780 nuclear counterstain, #FP150100KT, Akoya Biosciences

Validation

NF1
Representative immunohistochemistry images of NF1 staining in the current study are shown in Supplementary Information Figue 3. From the manufacturer's website: "Anti-Neurofibromin C, clone NFC, Cat. No. MABE1820, is a highly specific mouse monoclonal antibody that targets Neurofibromin and has been tested for use in Immunohistochemistry (Paraffin). Immunohistochemistry (Paraffin) Analysis: A representative lot detected Neurofibromin C in Immunohistochemistry applications (Rossi, S., et. al. (2018). Mod Pathol. 31(1):160-168). Western Blotting Analysis: A representative lot detected Neurofibromin C lysate from human NF1 +/+ cells, but not in -/- cells or NF1+/+ cells transfected with siRNA targeting neurofibromin (Reuss, DE.,et al., (2014). Acta Neuropathol. 127(4); 565-572)." https://www.sigmaaldrich.com/AU/en/product/mm/mabe1820#product-documentation

Immunofluorescence (IF) markers
All immunofluorescence markers used in this study are reported with full antibody metadata, including antigen target, clone, species, catalog number, antigen retrieval conditions, blocking reagents, antibody dilutions, detection polymers, and fluorophores (Supplementary Table S26). Antibodies were selected based on prior validation in FFPE tissue and established use in multiplex IF applications. Sequential multiplex staining was performed using tyramide signal amplification with Opal fluorophores, with heat-mediated antigen retrieval between staining cycles to prevent antibody cross-reactivity. Pan-cytokeratin staining was included in all panels to delineate tumor epithelium.
Staining specificity and signal quality were verified through iterative optimization and visual inspection during panel development. Automated cell detection and phenotyping were performed using QuPath (v0.2m2), with extensive manual training and validation to ensure accurate cell classification and compartmentalization into epithelial and stromal regions. Marker-positive cells were defined based on co-expression rules specified a priori, and $CD4^+$ T cells were operationally defined as $CD3^+CD8^-$ cells, consistent with prior reports (https://doi.org/10.1158/1078-0432.CCR-20-4394). All IF-derived quantitative data used in downstream analyses were generated using identical scoring parameters across samples.

Plants

Seed stocks	*Report on the source of all seed stocks or other plant material used. If applicable, state the seed stock centre and catalogue number. If plant specimens were collected from the field, describe the collection location, date and sampling procedures.*
Novel plant genotypes	*Describe the methods by which all novel plant genotypes were produced. This includes those generated by transgenic approaches, gene editing, chemical/radiation-based mutagenesis and hybridization. For transgenic lines, describe the transformation method, the number of independent lines analyzed and the generation upon which experiments were performed. For gene-edited lines, describe the editor used, the endogenous sequence targeted for editing, the targeting guide RNA sequence (if applicable) and how the editor was applied.*
Authentication	*Describe any authentication procedures for each seed stock used or novel genotype generated. Describe any experiments used to assess the effect of a mutation and, where applicable, how potential secondary effects (e.g. second site T-DNA insertions, mosiacism, off-target gene editing) were examined.*
